# Variation in zoo diets, offerings of leafy browse, and body condition scores in Matschie’s tree kangaroos (*Dendrolagus matschiei*) and their associations with gut microbiome composition

**DOI:** 10.7717/peerj.20875

**Published:** 2026-02-19

**Authors:** Diana C. Koester, Maura R. Plocek, Ellen S. Dierenfeld, Katherine R. Amato, Noah T. Dunham

**Affiliations:** 1 Biology, Case Western Reserve University, Cleveland, Ohio, United States; 2 Conservation and Science, Cleveland Metroparks Zoo, Cleveland, Ohio, United States; 3 Zootrition Animal Nutrition Consulting, St. Louis, Missouri, United States; 4 Anthropology, Northwestern University, Evanston, Illinois, United States

**Keywords:** Tree kangaroo, Diet, Gut microbiome, Gastrointestinal health

## Abstract

Recommended zoo diets for the arboreal folivore, the Matschie’s tree kangaroo (*Dendrolagus matschiei*; TK) were recently found to be much lower in fiber and higher in starch than wild diet items for this species. In contrast to wild animals, zoo-housed TKs are ~30–40% higher in body mass, known to suffer from infections indicating immune dysfunction, and exhibit various reproductive issues. These problems may have ties to gut microbiome dysbiosis related to differences in diet between captive and wild individuals, but to date, the microbiome of TKs has not been explored. This study aimed to (1) quantify the macronutrient intake of zoo-housed TKs and compare the intake of over-conditioned animals to those at ideal body condition, and (2) examine gut microbial communities for any differences associated with TK macronutrient intake, leafy browse offerings, or body condition. Detailed diet intake information (*n* = 31 individuals at 16 facilities accredited by the Association of Zoos and Aquariums) was collected for approximately 1 week at two different time points (*i.e*., July–September and January–March). Body condition scores were recorded by primary caregivers or veterinary staff no more than one month from each diet intake week. Fecal samples (*n* = 57) were collected concurrently during the diet recording weeks. We used 16S rRNA gene amplicon sequencing to examine TK gut microbiota. We found that animals assigned over-conditioned body scores consumed significantly more kilocalories compared to animals assigned an ideal body condition score. These differences were driven primarily by significantly greater crude protein and starch intake in over-conditioned TKs. TKs offered high and intermediate amounts of leafy browse exhibited substantially different fecal microbial communities compared to animals offered low or no browse. Our results indicate that formulation of diets for zoo-housed TKs, similar to other folivores, should closely resemble the macronutrient and caloric values of wild counterparts to encourage ideal body condition and promote gastrointestinal health. Future research should examine the gut microbiota of free-ranging TKs and assess how different species of leafy browse impact TK gut microbiota.

## Introduction

Recently, wildlife biologists have begun to realize the potential of investigations of the gut microbiome to profoundly advance our knowledge of threatened and endangered species health, host-microbiome coevolution, and conservation (Trevelline et al., 2019; Chong et al., 2020; Diaz & Reese, 2021; Dallas & Warne, 2023). Research on a diverse array of wildlife species has revealed overwhelming support for the influence of extrinsic environmental factors on the concentration and composition of organisms within the gut (reviewed in Trevelline et al. (2019)). For wild animals under human care, alterations and potential dysbiosis of the microbiome have been found to be driven by elements of the captive environment, such as administration of antibiotics (Plummer et al., 2005; Dahlhausen et al., 2018) and changes to species’ natural diets (Chong et al., 2020; Clayton et al., 2016; Kohl, Skopec & Dearing, 2014; Wasimuddin et al., 2017; McKenzie et al., 2017). Dietary specialists, and particularly those with a folivorous diet in the wild, appear to be highly susceptible to gut microbiome perturbations and dysbiosis when under human care (Chong et al., 2020; Kohl, Skopec & Dearing, 2014; Dallas & Warne, 2023). This is likely due to captive folivore diets differing significantly in macronutrients, such as lower fiber, compared to that of wild individuals (Clayton et al., 2016; Nakamura et al., 2011; Frankel et al., 2019). When the opposite is true and wild and zoo-housed folivores are fed similar diets due to strict dietary requirements for example, such as in the case of koalas (*Phascolarctos cinereus*), the gut microbiomes of captive and wild animals are remarkably similar (Chong et al., 2020; Alfano et al., 2015; Shiffman et al., 2017).

One such zoo-housed, arboreal folivore, the Matschie’s tree kangaroo (or Huon tree kangaroo; *Dendrolagus matschiei*; TK), is native only to remote cloud forests in the Huon penninsula of Papua New Guinea and is classified as Endangered by the IUCN with a decreasing population estimated to be 2,500 mature individuals (Ziembicki & Porolak, 2016). Due to the small remaining population size and secretive nature of this species, field scientists have only recently been able to uncover details of its natural diet (Dierenfeld et al., 2020). Wild TKs were found to consume a diet much higher in fiber and lower in starch and soluble sugars than typical *ex situ* diets for this species (Dierenfeld et al., 2020). Standard components of the diet of zoo-housed individuals are grain-based commercial biscuits, various leafy greens, a wide variety of fruits and vegetables, and some amount of locally available leafy browse (Carlyle-Askew, 2018). Although most local browse closely resemble the nutrient composition described for the diet of wild TKs (Dierenfeld, Bezjian & Dabek, 2021), browse is not typically fed out in sufficient quantities to be considered a primary diet item in North American zoos holding this species. Recommendations for captive diets have been updated recently, however, based on the preliminary findings of daily total energy requirements for two adult TKs in a zoo setting. TK energy expenditure was considerably lower than expected, based on body size and phylogeny, making this species particularly prone to overfeeding in human care (Dunham et al., 2022). In fact, in contrast to free-ranging individuals, zoo-housed TKs are, on average, ~30–40% higher in body mass than individuals living in the wild (Travis, Watson & Dabek, 2012). Captive TKs are also known to suffer from infections of *Mycobacterium avium* complex bacteria, an indication of immune dysfunction (Travis, Watson & Dabek, 2012; Buddle & Young, 2000; Blessington et al., 2021a) and the captive population remains unsustainable due to various reproductive issues (Blessington et al., 2021b). Each of these problems may have distinct ties to gut microbiome dysbiosis related to differences in diet between captive and wild individuals, but to date, the microbiome of TKs has not been explored, much less its relationship to diet or other environmental factors.

To address the gaps in current knowledge, the aims of this study were twofold. First, building on preliminary diet surveys requesting lists of items that are fed out, we sought to quantify the macronutrient consumption of TKs across zoos accredited by the Association of Zoos and Aquariums (AZA), and compare the macronutrient intake profiles of animals at ideal body condition to those that are assessed to be over-conditioned. We predicted that over-conditioned animals would consume significantly more metabolizable energy (*i.e*., kilocalories or kcal) than animals at ideal body condition. Secondly, we examined gut microbial communities of adult TKs in the AZA population for any differences associated with macronutrient intake, leafy browse offerings, or body condition. We predicted that increased fiber intake and increased leafy browse offerings would be consistent with greater bacterial diversity and increased relative abundance of fiber degrading bacteria and that over-conditioned animals would have decreased bacterial diversity. Our analyses will ultimately facilitate better understanding of the relationship between specific diet components and gut microbial profiles, thus informing diet recommendations for *ex situ* TKs targeted to improve population sustainability and health.

## Materials and Methods

### Study subjects

The study was conducted in strict accordance with recommendations in the Guide for the Care and Use of Laboratory Animals of the National Institutes of Health. All research protocols reported in this manuscript were reviewed and approved by Cleveland Metroparks Zoo’s Scientific Review Committee (CS2021-018). All samples were collected non-invasively (feces), therefore not requiring IACUC approval or special permits. Matschie’s TKs (*n* = 31) were housed at 16 institutions in North America, all accredited by AZA and accounting for over 75% of the entire population of TKs in AZA facilities. All animals included were captive-born, housed singly, and managed according to protocols and husbandry guidelines established by the TK Species Survival Plan (SSP). Study animals were between 1.4 and 20.5 years of age (mean ± standard deviation [SD], 9.9 ± 5.7 years). Data and samples from a total of 14 males and 17 females were used for the study, totaling 57 separate instances of week-long diet intake data, each paired with a single, concurrent fecal sample. Data and feces from all but five animals were collected twice in the same year, about 6 months apart, to reflect seasonal differences in browse offerings (see details in following section). Data were included from females only if they were known not to be pregnant or lactating, or in the early stages of lactation (less than 5 months), to ensure minimal impact on diet intake. For data analyses including body condition scoring categories, four females were excluded due to a lack of available body condition scoring information, resulting in 27 total animals analyzed in this subset, and 50 fecal samples with concurrent diet intake data. For the body condition score subset, minimum age was slightly higher at 2.9 years, and is therefore more reflective of fully adult animals, with little to no additional growth or development expected to occur (Blessington & Steenberg, 2007).

### Dietary intake and browse offerings

We quantified the food and macronutrient intake of TKs from August 2021 to August 2022. We aimed to sample the diet intake of individual TKs for approximately 1 week (*i.e*., 5–7 consecutive days) at two different time points (*i.e*., July–September and January–March) to account for potential seasonal differences in browse offerings. All diet components, aside from leafy browse, are available year-round for TKs in AZA zoos. Leafy browse, however, can be offered at intermediate or high quantities during the growing seasons of late spring, summer, and early to mid-fall for most regions, but drops to little or no browse offered during winter season. Therefore, two sampling efforts, taking place at the peak time of each of the two variations in offered diets, are sufficient to capture any seasonal differences in TK diets. Because the study animals were housed and fed separately, it was possible to determine individual food and macronutrient intake. With the help of animal care staff, we recorded the mass of each food item (*i.e*., commercial pellets, leafy greens, fruits, vegetables, *etc*.,) offered per day. After controlling for moisture lost overnight based on experimental trials, we subtracted the mass of each food item remaining to estimate the mass of various food items consumed (Dunham et al., 2022). Daily macronutrient intake was quantified by multiplying the mass of each food item consumed by the nutrient composition of each food item.

We used published nutrient composition values from Schmidt et al. (2005) for the majority of food items (*e.g*., leafy greens, fruits, and vegetables) offered to our study subjects. Food items not reported by Schmidt et al. (2005) were sent to Dairy One Forage Laboratory (Ithaca, NY, USA) for macronutrient analyses following standardized procedures for ash (Association of Official Agricultural Chemists (AOAC) 942.05), crude protein (CP: AOAC 990.03), neutral detergent fiber (NDF: Van Soest, Robertson & Lewis, 1991), starch (Xylem YSI Life Sciences Application Note Number 222LS-02), soluble sugars (SC: Hall et al., 1999), and crude fat (CF: AOAC 954.02). Total nonstructural carbohydrate (TNC) was estimated *via* subtraction: TNC = [100 − (%Ash + % CP + % NDF + % CF)]. We quantified daily metabolizable energy (ME) intake by multiplying standard physiological fuel values for CP (4 kcal/g), TNC (4 kcal/g), NDF (3 kcal/g), and CF (9 kcal/g) (Van Soest, 1994). We adjusted the energy intake value attributable to NDF by multiplying the value by the mean apparent NDF digestibility coefficient for *Dendrolagus matschiei* (*i.e*., 60.8%) reported by Dunham et al. (2022). We then summed ME intake from CP, TNC, NDF, and CF to estimate daily metabolizable energy intake.

Due to the difficulties in accurately quantifying leafy browse consumption, we opted to categorically define leafy browse offerings based on both the amount and frequency in which it was offered: none = no browse offered during sampling period; low = ~1–4 branches (~1 m in length) offered two to four times per week, intermediate = ~3–6 branches offered five to seven times per week, and high = ~8–12+ branches offered daily.

### Body condition scoring

**Figure 1:**
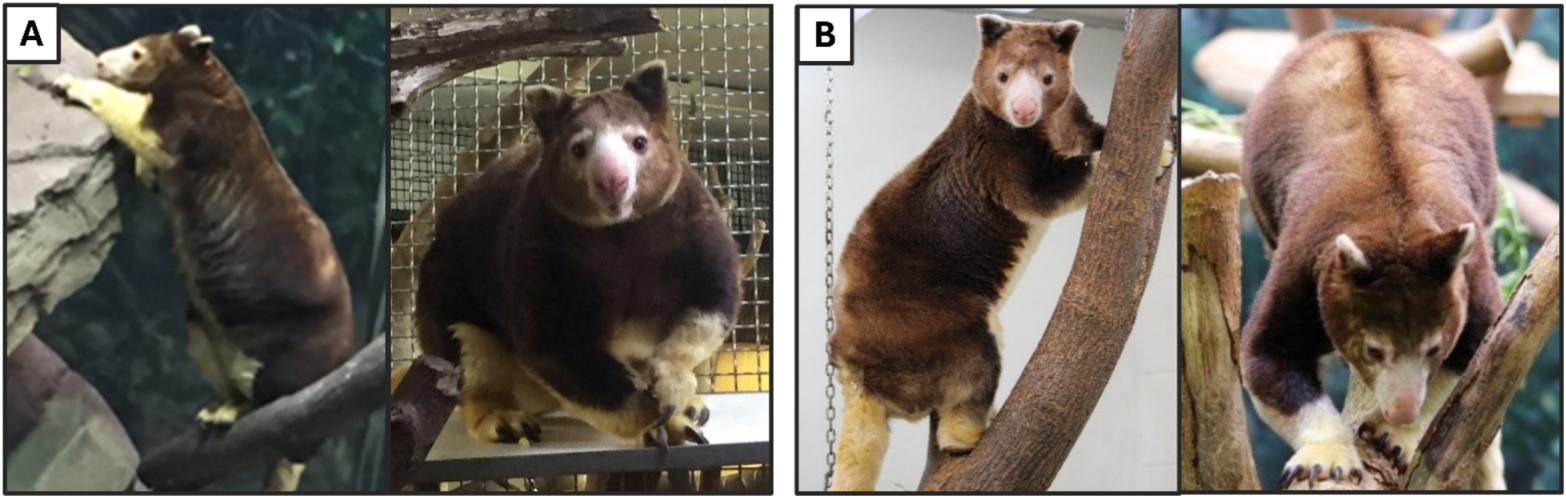
Photographs of Matschie’s tree kangaroos (*Dendrolagus matschiei*) included in this study and body condition scored according to available criteria and categorized as over-conditioned (A) and ideal (B). Photos by D. Koester and N. Dunham. Note pendulous fat deposits on the chest and shoulders and lack of clear neck definition in the over-conditioned animal, as well as significant fat accumulation on the side of the abdomen, just above the hip, that is not present in the ideally conditioned animal.

Body condition scores (BCS) were assigned at individual home institutions by veterinary staff or primary caregivers familiar with available TK BCS parameters shared by AZA SSP group leaders (Supplemental Material). Scoring was done by tactile or visual assessment within 1 month of each instance of diet intake data recording and fecal sample collection. Due to the lack of any animals in the study scored below ideal and the small sample size of this population, BCS information was collapsed into two categories for analysis, ideal and over-conditioned (for examples, see Fig. 1; for full BCS, see Fig. S1). As mentioned previously, all data from four females were excluded during analyses of BCS because recent scores were not able to be obtained from these animals.

### Fecal sample collection and processing

We collected one fecal sample near the end of each diet recording week for a given individual (*i.e*., on day 5, 6, or 7) to ensure that fecal microbiota data would be temporally linked with diet intake data. Fecal samples were collected as soon as possible after voiding (*i.e*., maximum time after defecation of 4 h). Samples were submerged in 96% ethanol and stored in sterile containers at ambient temperature and then shipped to Cleveland Metroparks Zoo. We extracted microbial DNA within 4 months of sample collection using the Qiagen DNeasy Powersoil Pro DNA extraction kit (Germantown, MD, USA) with provided protocol.

DNA extracts were stored at −80 °C and then shipped to the Amato Lab at Northwestern University for further processing. We amplified the V4–V5 region of the 16S ribosomal RNA (rRNA) gene using previously published methods (Mallott & Amato, 2018). Briefly, we performed a two-step polymerase chain reaction (PCR; Step 1: 515F/926R primers with CS1/CS2 linkers added, Step 2: Fluidigm Access Array barcoding primers) using Phusion Taq DNA polymerase. The resulting amplicons were sequenced at the DNA Services Facility at Rush University Genomics and Microbiome Core on the Illumina MiSeq platform.

### 16S sequence processing

Sequencing yielded 2,645,691 total reads with an average of 46,415 reads per sample before quality filtering. QIIME2 (version 2023.2) was used to process raw reads (Bolyen et al., 2019). DADA2 algorithms were used to trim, quality-filter, denoise, and dereplicate sequence data, merge paired-end reads, and cluster amplicon sequence variants (ASVs) (Callahan et al., 2016). Singleton reads in this analysis were not treated as separate ASVs, because the denoising procedure required at least two reads to support a unique sequence before accepting it as a variant. We removed chimeric, chloroplast, and mitochondrial sequences, resulting in a total feature frequency of 1,130,422 and an average of 18,685 features per sample, while ASV’s assigned to archaeal or other non-bacterial domains were retained rather than filtered out. Taxonomy was assigned using SILVA database (release 138; Quast et al., 2012). All samples were rarefied to 10,000 reads per sample, resulting in five samples being removed from further analyses. Extraction blanks and PCR no-template controls were processed through the full pipeline and produced zero reads after quality filtering, thus rendering standard contaminant removal steps unnecessary. Microbial sequence data are accessible *via* NIH NCBI BioProject: PRJNA1252074 and relative abundance of different bacterial taxa are provided as an interactive hierarchical Krona chart (see Supplemental Material).

### Statistical analyses

We first examined dietary intake and variation across the sample population by generating the mean, standard deviation, and coefficient of variation (CV) for each macronutrient based on daily intake. Linear mixed models were used to assess the effect of BCS category (ideal *vs*. over-conditioned) on total daily kcal intake, total daily dry matter intake, and daily macronutrient intake (*i.e*., CP g, NDF g, starch g, SC g, and CF g). Sex was included as a covariate to control for potential differences in amount of food consumed between males and females due to increased body size in the former. Age was also included as a covariate to control for potential age-related differences in dietary intake. Individual was included as a random factor. A generalized linear mixed model was used to assess the relationship between BCS category and browse category (*i.e*., none, low, intermediate, or high) with individual animal included as a random factor. Statistical analyses were conducted using R Statistical Software (v4.3.3; R Core Team, 2024) including add-on packages: lme4 (Bates et al., 2014), lmerTest (Kuznetsova, Brockhoff & Christensen, 2017), and multpois (Wobbrock, 2024).

We generated microbial community richness and diversity in each sample using four alpha diversity indices: observed ASV features, evenness, Shannon index, and Faith’s phylogenetic diversity index. Observed ASV features quantify the number of unique Amplicon Sequence Variants (ASVs) within a sample, providing a measure of species richness without accounting for species abundance (Callahan, McMurdie & Holmes, 2017). Evenness (scored from 0 to 1) evaluates how uniformly individual microbial species are distributed within a sample, where high evenness reflects similar abundances, and low evenness indicates dominance by a few species (Magurran, 2013). The Shannon index integrates both richness and evenness, considering species counts and their relative abundances, with higher values corresponding to greater diversity (Kers & Saccenti, 2022). Finally, Faith’s (1992) phylogenetic diversity calculates the total branch length of the phylogenetic tree encompassing the species in a sample, thereby capturing the phylogenetic breadth of the community.

Linear mixed models were used to examine the effects of macronutrient intake (CP, NDF, starch, SC, and CF expressed as % of dry matter intake), browse category (none, low, intermediate, high), BCS category (ideal, over-conditioned), age, and sex on each alpha diversity index with individual TK included as a random/repeated variable. We quantified both unweighted and weighted UniFrac distances among all samples and visualized data using nonmetric multi-dimensional scaling. We measured beta-dispersion to evaluate inter-individual variation within groups (*i.e*. browse category and BCS) and tested for uniformity of dispersion. We then used permutational multivariate analysis of variance (PERMANOVA) to examine the effects macronutrient intake, browse category, body condition, age, and sex on score category on overall microbiome composition. Posthoc pairwise adonis tests with Bonferroni corrections for multiple comparisons were used to assess potential differences in beta diversity among different browse categories (Arbizu, 2020). Microbial composition of samples was assessed using QIIME 2 bioinformatics platform (Bolyen et al., 2019). We tested for significant associations between fixed factors of interest (*i.e*., macronutrient intake, browse category, and BCS) and differential microbial abundance, with individual included as random/repeated term, using the linear mixed models in MaAsLin2 package (Mallick et al., 2021). To improve statistical validity and control for false discovery, additional prevalence filtering was applied for differential abundance analyses to remove features with 100 reads or fewer and/or present in fewer than three samples, resulting in a mean of 153 ASVs (Cao et al., 2020; Zhou et al., 2023).

## Results

### Dietary intake, browse offerings, and BCS

**Figure 2:**
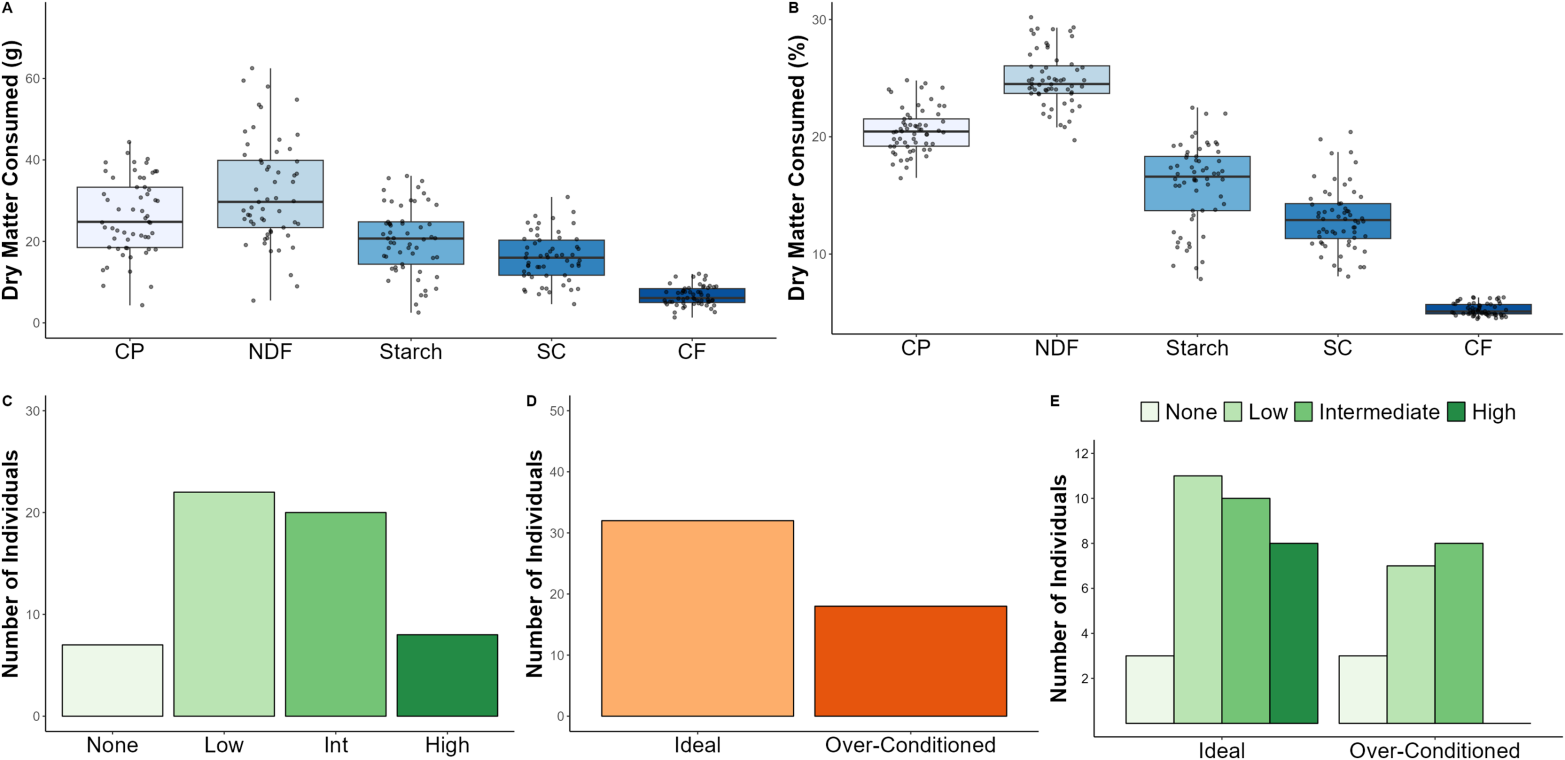
Dietary intake, browse category, and BCS data for North American zoo-housed Matschie’s tree kangaroos (*Dendrolagus matschiei*), including absolute dry matter consumed (A), relative proportions of dry matter consumed (B), number of individuals ass. There are seven diet intake weeks without an assigned BCS, hence the sample size discrepancy between panel C and panel E. DM, dry matter; CP, crude protein; NDF, neutral detergent fiber; SC, soluble carbohydrates; CF, crude fat.

Dry matter dietary intake averaged 127.8 ± 47.0 g and varied considerably across the study sample (CV = 36.7%; Table 1; Fig. 2A). Intake of different macronutrients was also highly variable (*i.e*., CV ranging from ~ 36–41%) with starch intake varying to the greatest degree across individuals. Daily average energy intake was estimated to be 436.5 ± 159.2 kcal (Table 1). When examining the relative proportions of macronutrients to dry matter intake, NDF was found to constitute the largest proportion of the diet (25.0 ± 2.4%), followed by CP (20.5 ± 1.9%), starch (15.9 ± 3.6%), SC (12.9 ± 2.6%), and CF (5.2 ± 0.5%) (Table 1; Fig. 2B). Once again, starch was the most variable component of the diet (CV = 22.5%); however, macronutrient intakes reported as relative proportions were considerably less variable (*i.e*., CV ranging from ~ 9–23%) than those reported as absolute (g) intake (Table 1).

**Table 1:** Dietary intake of North American zoo-housed Matschie’s tree kangaroos (*Dendrolagus matschiei; n* = 31 individuals, *n* = 57 analyzed diet intake instances).

	Daily intake (g)			DM intake (%)		
Intake variable	Mean	Stdev	CV (%)	Mean	Stdev	CV
DM	127.8	47.0	36.7	–	–	–
CP	26.2	9.4	35.8	20.5	1.9	9.3
NDF	32.2	12.8	39.8	25.0	2.4	9.5
Starch	20.4	8.3	40.5	15.9	3.6	22.5
SC	16.0	5.9	36.6	12.9	2.6	20.4
CF	6.6	2.4	36.0	5.2	0.5	10.3
Kcal	436.5	159.2	36.5	–	–	–

**Note:**

DM, dry matter; CP, crude protein; NDF, neutral detergent fiber; SC, soluble carbohydrates; CF, crude fat; Stdev, standard deviation; CV, coefficient of variation.

We assigned a leafy browse offering category to each of the 57 weekly diet intake timepoints based on the amount and frequency in which browse was offered. For example, the browse category for an animal could be classified as “high” during one timepoint, while the browse category for the same individual could be classified as “low” during a different timepoint. Across the study period, browse categories were classified as none (12.3%), low (38.6%), intermediate (35.1%), or high (14.0%) (Fig. 2C).

We assessed BCS throughout the study and categorized individuals as ideal *vs*. over-conditioned. Animals were recorded as ideal (64.0%) and over-conditioned (36.0%), respectively (Fig. 2D).

### Relationships among BCS, diet intake, and browse offerings

Compared to females, males consumed significantly more DM g (*p* = 0.02), CP (*p* = 0.009), NDF (*p* = 0.02), SC (*p* = 0.03), CF (*p* = 0.02), and total kcal (*p* = 0.02) (Table S1). Age did not significantly affect diet intake (Table S2). BCS category had a significant effect on DM g, CP g, starch g, and total daily kcal. Over-conditioned animals consumed more DM g and total kcal compared to individuals of ideal BCS (*p* = 0.05 and *p* = 0.04, respectfully). The increased dry matter and total caloric intake of over-conditioned animals was driven by elevated CP and starch intake compared to individuals of ideal BCS (*p* = 0.04 and *p* = 0.03, respectfully). All other macronutrient intake variables were consistent between BCS categories (Table 2).

**Table 2:** Effects of BCS category on dietary intake and pairwise comparisons of estimated marginal means between ideal and over-conditioned BCS categories in North American zoo-housed Matschie’s tree kangaroos (*Dendrolagus matschiei*).

Parameter	F-value	df	*P*-value	Ideal	Over-conditioned
DM g	4.3	1, 22.7	**0.05**	116.7^a^ (97.1–136.2)	147.5^b^ (123.7–171.2)
CP g	4.8	1, 22.0	**0.04**	24.0^a^ (20.4–27.6)	30.0^b^ (25.6–34.4)
NDF g	2.5	1, 23.0	0.13	29.9^a^ (24.5–35.3)	36.5^a^ (29.9–43.0)
Starch g	5.5	1, 21.6	**0.03**	17.8^a^ (14.1–21.5)	24.4^b^ (19.9–28.8)
SC g	2.3	1, 22.9	0.14	14.9^a^ (12.2–17.5)	18.0^a^ (14.7–21.2)
CF g	3.3	1, 23.3	0.08	6.2^a^ (5.1–7.2)	7.6^a^ (6.3–8.8)
Kcal	4.7	1, 22.6	**0.04**	405.8 ^a^ (339.2–472.4)	514.8 ^b^ (434.0–595.7)

**Note:**

Parentheses contain 95% confidence intervals. Shared superscripts within a row indicate that estimated marginal means were not significantly different. DM, dry matter; CP, crude protein; NDF, neutral detergent fiber; SC, soluble carbohydrates; CF, crude fat; df, degrees of freedom; df, degrees of freedom. Bold *p*-values indicate statistically significant results.

We intended to examine the effect of BCS on browse category using generalized linear mixed models; however, the model failed to converge likely due to the nature of the dataset. That is, the number of individuals characterized by browse categories of none, low, and intermediate were generally similar between the ideal and over-conditioned BCS categories; however, all eight individuals characterized by the high browse category belonged to the ideal BCS category (Fig. 2E).

### Effects of macronutrient intake, browse category and BCS on bacterial alpha diversity

**Figure 3:**
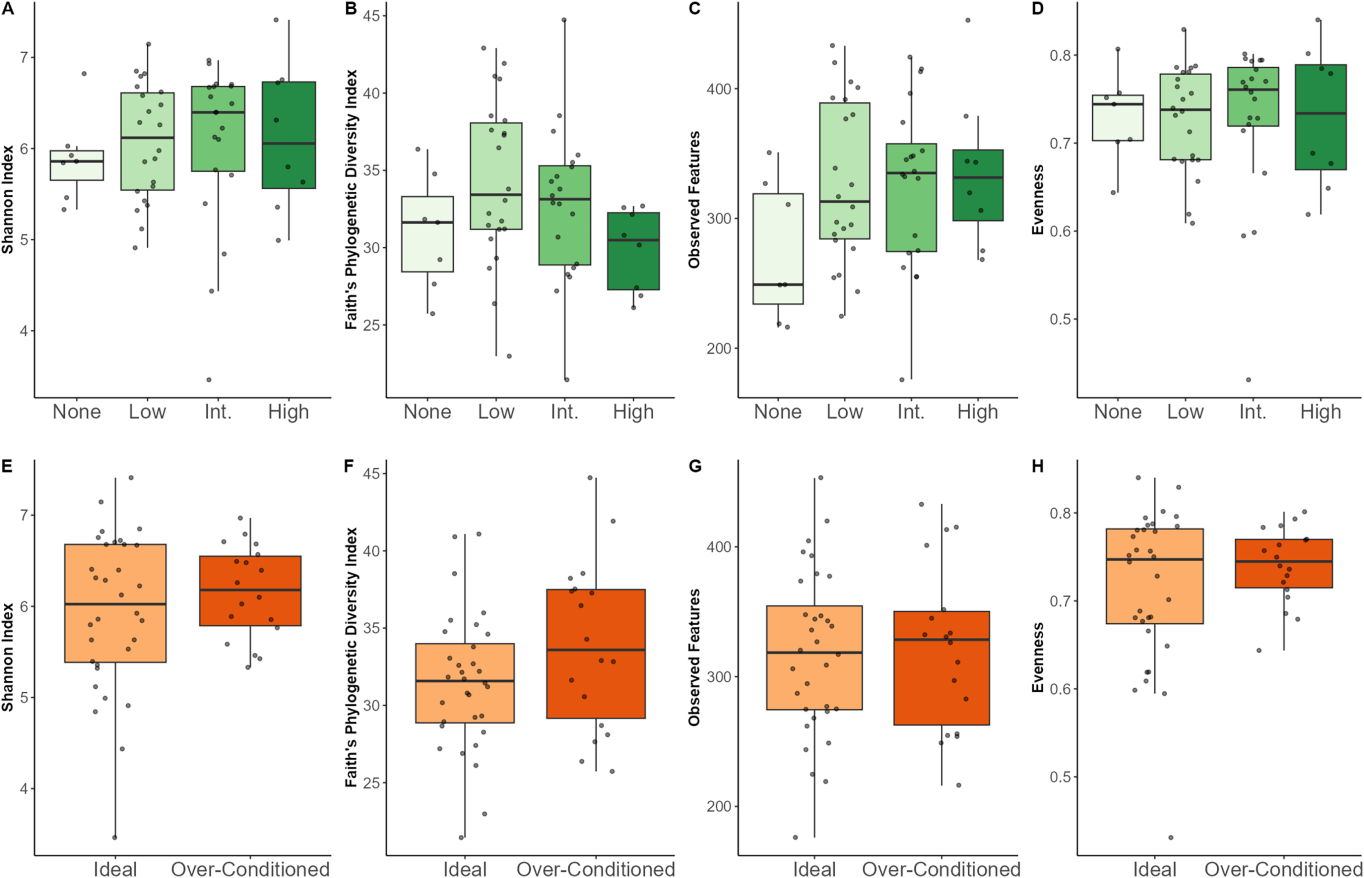
Alpha diversity indices for North American zoo-housed Matschie’s tree kangaroos (*Dendrolagus matschiei*) by browse category (A–D) and BCS (E–H).

Regarding macronutrients, increased SC intake was associated with lower Shannon diversity (*p* = 0.02) and lower evenness (*p* = 0.007). Increased CF intake was associated with a greater number of observed features (*p* = 0.03) (Figs. S2–S5). Neither browse category nor BCS category significantly affected any of the alpha diversity metrics examined (Table 3). Though not statistically significant, individuals that were not offered leafy browse (*i.e*., browse category = none) exhibited the lowest diversity indices for Shannon diversity and observed features (Fig. 3).

**Table 3:** Effects of macronutrient intake, browse category, BCS category, sex, and age on alpha diversity indices in North American zoo-housed tree kangaroos (*Dendrolagus matschiei*).

	F-value	df	*p*-value	
**Shannon index**				
CP	1.20	1, 24.6	0.28	
NDF	0.25	1, 21.2	0.62	
Starch	2.53	1, 22.5	0.13	
SC	5.90	1, 33.9	**0.02**	
CF	3.95	1, 28.4	0.06	
Browse category	1.12	3, 29.6	0.35	
BCS	0.57	1, 17.8	0.46	
Sex	1.45	1, 20.7	0.24	
Age	0.00	1, 17.8	0.98	
**Faith’s PD index**				
CP	0.10	1, 26.6	0.75	
NDF	0.04	1, 22.5	0.84	
Starch	0.38	1, 24.0	0.54	
SC	0.02	1, 35.1	0.89	
CF	0.34	1, 30.5	0.56	
Browse category	2.31	3, 32.6	0.09	
BCS	1.73	1, 18.1	0.20	
Sex	2.32	1, 18.0	0.15	
Age	0.01	1, 20.8	0.92	
**Observed features**				
CP	0.21	1, 23.9	0.65	
NDF	0.07	1, 20.7	0.79	
Starch	3.91	1, 21.9	0.06	
SC	0.13	1, 33.4	0.72	
CF	5.29	1, 27.7	**0.03**	
Browse category	1.76	3, 28.7	0.18	
BCS	0.32	1, 17.5	0.58	
Sex	0.78	1, 17.5	0.39	
Age	0.71	1, 20.4	0.41	
**Evenness**				
CP	2.23	1, 23.8	0.15	
NDF	0.49	1, 20.5	0.49	
Starch	1.67	1, 21.7	0.21	
SC	8.28	1, 33.4	**0.007**	
CF	2.89	1, 27.6	0.10	
Browse category	1.30	3, 28.7	0.29	
BCS	0.57	1, 17.2	0.46	
Sex	1.44	1, 17.2	0.25	
Age	0.05	1, 20.1	0.83	

**Note:**

CP, crude protein; NDF, neutral detergent fiber; SC, soluble carbohydrates; CF, crude fat; BCS, body condition score; df, degrees of freedom. Bold p-values indicate statistically significant results.

### Effects of macronutrient intake, browse category, and BCS on bacterial beta diversity

Beta dispersion analyses revealed that there was a significant difference in group dispersion among the browse categories in the unweighted UniFrac (*p* < 0.001). The high browse category had lower inter-individual variation than the none (*p* = 0.02) and low browse categories (*p* < 0.001) (Fig. S5). The intermediate browse category also exhibited lower variation than the low browse category (*p* = 0.04). Beta dispersion of BCS categories for unweighted UniFrac were not significant. Neither browse category nor BCS category differed significantly in their beta dispersion (Fig. S5).

**Figure 4:**
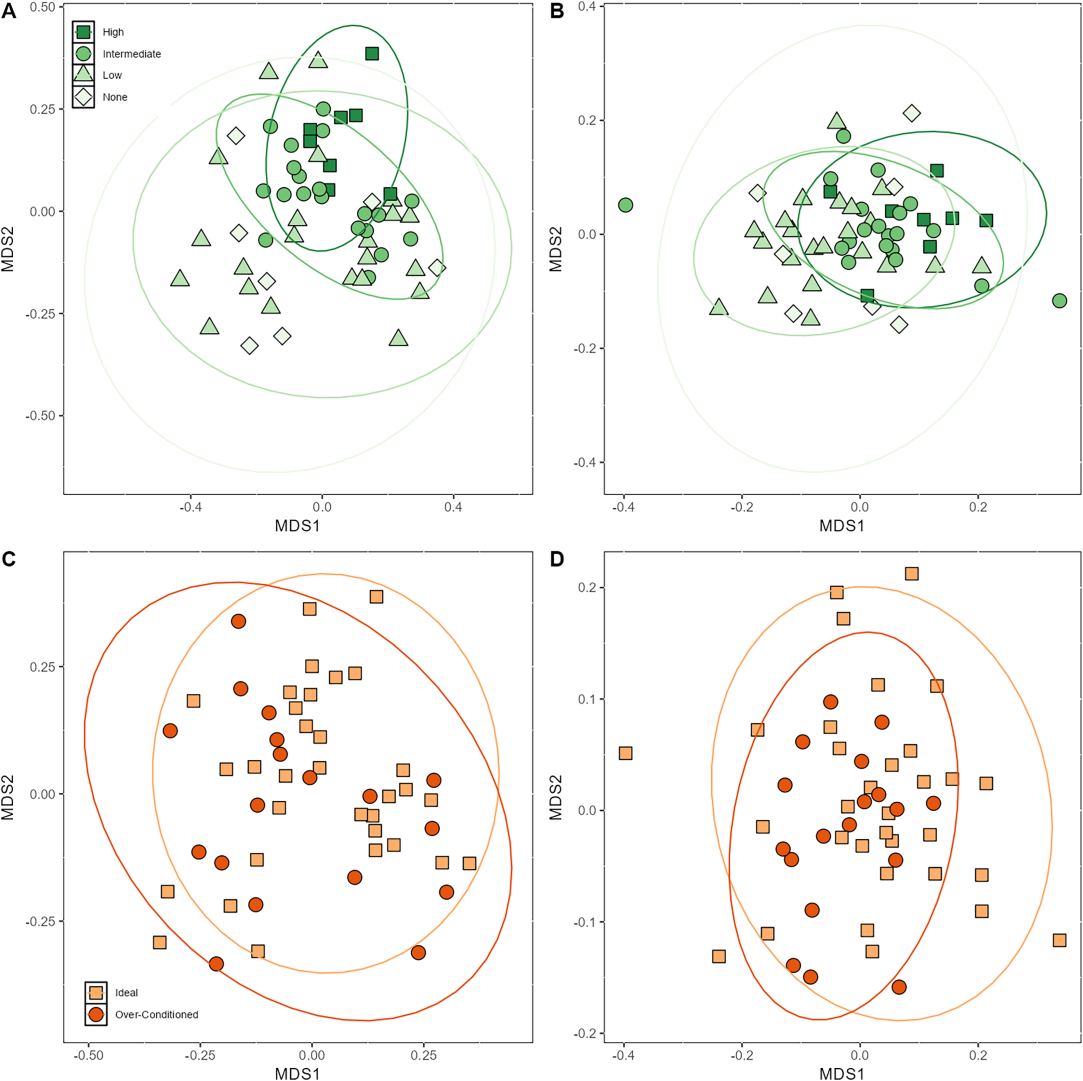
Nonmetric multidimensional scaling (NMDS) plot showing (A) unweighted (B) and weighted UniFrac metrics demonstrating clustering of samples by browse category and (C) unweighted (D) and weighted UniFrac metrics demonstrating clustering of samples by BCS ca.

Based on PERMANOVA analyses, the proportions of macronutrients ingested impacted bacterial beta diversity indices. Unweighted UniFrac was significantly affected by CP (*p* = 0.03) and CF content (*p* = 0.04) (Table 4). Browse category also significantly affected unweighted UniFrac (*p* < 0.001) (Table 4; Fig. 4A). Posthoc pairwise adonis tests revealed that the high browse category differed significantly from the none (*p* = 0.02), low (*p* = 0.006), and intermediate (*p* = 0.01) browse categories. Body condition category, sex, and age did not significantly impact unweighted UniFrac.

**Table 4:** PERMANOVA results showing the effect of macronutrient intake, browse category, BCS category, sex, and age on unweighted and weighted UniFrac metrics of community similarity.

Parameter	Unweighted UniFrac	Weighted UniFrac
CP	F = 1.41, r^2^ = 0.03, ***p* = 0.03**	F = 1.03, r^2^ = 0.02, *p* = 0.39
NDF	F = 1.23, r^2^ = 0.02, *p* = 0.10	F = 0.72, r^2^ = 0.01, *p* = 0.73
Starch	F = 1.17, r^2^ = 0.02, *p* = 0.14	F = 1.22, r^2^ = 0.02, *p* = 0.25
SC	F = 1.27, r^2^ = 0.02, *p* = 0.08	F = 0.93, r^2^ = 0.02, *p* = 0.50
CF	F = 1.51, r^2^ = 0.03, ***p* = 0.02**	F = 1.407, r^2^ = 0.02, *p* = 0.15
Browse category	F = 1.54, r^2^ = 0.09, ***p* < 0.001**	F = 2.26, r^2^ = 0.12, ***p* = 0.001**
BCS category	F = 0.92, r^2^ = 0.02, *p* = 0.62	F = 1.36, r^2^ = 0.02, *p* = 0.16
Sex	F = 1.26, r^2^ = 0.02, *p* = 0.07	F = 1.56, r^2^ = 0.03, *p* = 0.09
Age	F = 1.24 r^2^ = 0.02, *p* = 0.10	F = 1.63, r^2^ = 0.03, *p* = 0.08

**Note:**

CP, crude protein; NDF, neutral detergent fiber; SC, soluble carbohydrates; CF, crude fat; BCS, body condition score.

Bold *p*-values indicate statistically significant results.

Browse category also significantly impacted weighted UniFrac (*p* = 0.001) (Fig. 4B). Posthoc pairwise adonis tests revealed that the high browse category differed significantly from the low (*p* = 0.006) browse category and the intermediate browse category also differed significantly from the low browse category (*p* = 0.03). None of the macronutrients ingested nor BCS significantly impacted weighted UniFrac beta diversity indices (Table 4; Figs. 4C and 4D).

### Effects of diet intake, browse category and BCS on bacterial abundance

**Figure 5:**
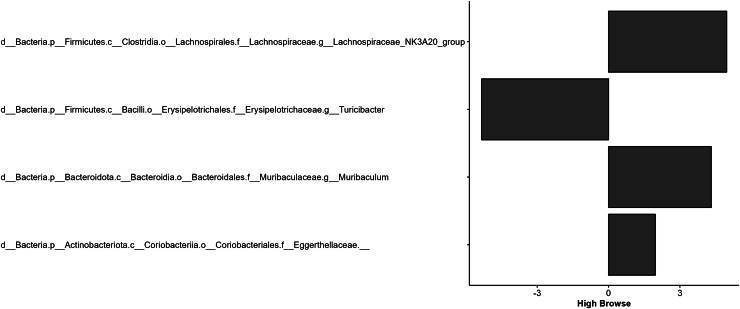
Effect of the high browse category relative to the no browse category on the differential abundance of microbial taxa. More positive values indicate enrichment, and more negative values indicate depletion of a particular taxa.

Macronutrient intake, BCS, age, and sex did not significantly impact the relative abundance of bacterial taxa; however, browse category significantly impacted the relative abundance of four bacterial taxa (Fig. 5). Enrichment (positive values) and depletion (negative values) of bacterial taxa are relative to the no browse reference condition. Significant differences were driven by the high browse category. The high browse condition was associated with decreased relative abundances of the genus *Turicibacter* (*p* < 0.001) and with increased relative abundances of the genus *Muribaculum* (*p* < 0.001), the genus classified as Lachnospiraceae_NK3A20_group (*p* = 0.003), and an unidentified taxon belonging to the family Eggerthellaceae (*p* = 0.002).

## Discussion

In an effort to advance our understanding of what TKs in AZA zoos are currently eating and clarify diet connections to body condition and specific changes in the gut microbiome for this species, we aimed to (1) quantify the macronutrient consumption of TKs across zoos, (2) compare the macronutrient intake profiles of animals at ideal body condition to those that are over-conditioned, and (3) examine gut microbial communities for any differences associated with macronutrient intake, leafy browse offerings, or body condition.

While previous surveys have already established a high degree of variability in diets offered to TKs in human care (Carlyle-Askew, 2018), our study is the first to fully capture detailed daily diet intake data for over 75% of the AZA population of TKs. Lowest coefficients of variation in macronutrient categories were as high as 35.8%, meaning that the macronutrients that were the most similar across animals and institutions still differed from the overall mean by more than a third. Of additional note is the consistency in the extreme variability observed in both what different zoos are offering TKs, as well as in what we found TKs at different institutions are choosing to consume, particularly in terms of overall kcal per day. This indicates the inability of some individual animals to self-regulate diet intake to match daily energy expenditure requirements. Our finding of over-conditioned animals exhibiting an average daily intake of 514.8 kcal, or more than 100 kcal higher than the daily average for animals of ideal BCS (*i.e*., ~27% increase) further emphasizes this point. Dierenfeld et al. (2020) found that food items consumed by free-ranging Matschie’s TKs averaged 10.9% CP and <1% starch by dry matter, while we reported dry matter intake of zoo-housed TKs to be 20.5% CP and 15.9% starch. Furthermore, though NDF intake did not differ significantly between animals at ideal *vs*. over-conditioned body scores, it is important to highlight the considerable discrepancy between the mean 25.0 ± 2.4% NDF dry matter intake of zoo-housed TKs and values in foods consumed by free-ranging TKs of 52.0 ± 13% NDF (Dierenfeld et al., 2020). These substantial disparities, particularly with regard to starch and NDF, result in more easily metabolizable energy and ultimately greater caloric intake in some zoo-housed TKs.

Given the challenges of accurately quantifying browse intake, leafy browse did not contribute to our estimates of daily DM, macronutrient, or caloric intake. In our previous study of two TKs, we estimated leafy browse to contribute ~174–230 kcal per day (Dunham et al., 2022); however, these two individuals received substantially more browse than any other individuals in the current study. While we acknowledge the limitation of our inability to quantify browse intake in this study, we do not expect browse consumption to substantially alter daily caloric intake for the majority of individuals in the study, and we expect the caloric discrepancy between individuals identified as over-conditioned *vs*. ideal to remain if caloric intake attributable to browse was included in the analyses. The daily intake averaging approximately 400 kcal for animals at an ideal BCS also agrees with previous work on this species, reporting the total energy expenditure of two adult animals to be approximately 300–400 kcal per day (Dunham et al., 2022). These animals were both in ideal body condition and maintained on diets containing a high proportion of leafy browse.

One limitation of the current analyses is the subjectivity of BCS assessments for this species and the relative recency of the established scoring chart descriptions for TKs (2017), making it difficult for zoo professionals to reach levels of inter-assessor reliability and intra-assessor consistency common in the agricultural industry (Evans, 1978). This subjectivity limited our ability to accurately compare diet intake and microbial community structure among animals differentiated by single points on the established BCS scale, as many individuals were reported as point ranges likely reflecting differences in opinion among staff members at each institution. As individuals tended to be consistently at or above ideal BCS, however, we chose to simply create those two categories for our analysis. The subjectivity may still have contributed to inaccuracies in the creation of our experimental groups though, and it therefore limited our ability to accurately test for changes in microbiome composition across individuals of differing BCS. Future efforts to more objectively measure body fat composition and obesity (*e.g*., deuterium oxide dilution; Dunham et al., 2022) could reveal shifts in the gut microbiome, such as an increased ratio of Firmicutes:Bacteriodes, a suspected biomarker for obesity in humans and other species (Ley et al., 2006; De Wit et al., 2012; Hildebrandt et al., 2009; De Filippo et al., 2010).

Browse is a crucial component of folivorous animal diets and is essential for maintaining animal welfare. Browse provisioning has been associated with increased foraging time (Birke, 2002; Cassella, Mills & Lukas, 2012; Lasky et al., 2021; Dunham et al., 2023; Fuller et al., 2018), decreased inactivity (Birke, 2002; Dunham et al., 2023), enhanced satiety (Smith, Remis & Dierenfeld, 2020), and a reduction in stereotypic or undesirable behaviors in certain taxa (Baxter & Plowman, 2001; Cassella, Mills & Lukas, 2012; Fuller et al., 2018; Bollnow, Podraza & Miller, 2025). However, it is challenging for zoos, particularly those in temperate climates, to provide year-round browse due to the financial and logistical constraints involved in selecting and sourcing appropriate browse items (reviewed in Ramont et al. (2024)). Consequently, many zoos provide intermediate or high quantities of browse seasonally, during the growing season when it is relatively easy to collect locally and then reduce quantities or completely omit browse during the non-growing season. In the current study, browse quantity was the only part of the diet reported by zoos to change over the course of a year, from the growing season to the winter, as all other diet items were readily available year-round. Sampling twice a year, in the peak of each season, was therefore sufficient to capture wholesale seasonal changes in TK diets. This approach does not consider potential differences in browse nutritional quality over the course of a growing season, however, and should be considered a limitation. Future gut microbiome investigations of TKs should include sampling efforts spread over the shoulder seasons as well to identify changes possibly associated with browse quality.

Browse quantity, particularly the high browse category, was associated with significant changes in the composition of the fecal microbiome. These changes were driven both by the presence and absence of specific taxa and their relative abundance, suggesting a substantial shift in the overall microbial community structure as opposed to a change in the abundance of a few dominant taxa. Although the effect sizes were statistically significant, they were fairly small, suggesting that browse provisioning accounted for a meaningful, but limited, share of microbiome variation.

*Muribaculum*, Lachnospiraceae, and Eggerthellaceae were enriched in the high browse category and are considered commensal bacteria. *Muribaculum* is a known fiber degrader that can cross-feed other bacteria, including Lachnospiraceae, to increase production of short-chain fatty acids that are critical in regulating the function of the intestinal barrier and immune response (Chung et al., 2020; Zhu et al., 2024). Previous research also found increased Muribaculaceae abundance in cows (*Bos taurus)* and mice (*Mus musculus*) fed diets high in fiber, resistant starch, and inulin (Wan et al., 2021). Lachnospiraceae, as a family, serves a key role in the gut microbiome by fermenting complex plant fibers into the short-chain fatty acids acetate and butyrate, thereby supporting host energy harvest and colonic health (Vacca et al., 2020). More specifically, Lachnospiraceae_NK3A20_group is a core taxon in herbivorous ruminants that not only contributes to SCFA production but also exhibits flexible hydrogen production and utilization, providing redundancy in metabolic pathways and enhancing the robustness of gut fermentation processes under varying environmental conditions (Kaminsky et al., 2023; Tovar-Herrera et al., 2025). Microbes in the Eggerthellaceae family hydrolyze ellagitannins, resulting in the production of urolithins that have strong antioxidant (Cerdá, Tomás-Barberán & Espín, 2005), anti-inflammatory (Larrosa et al., 2010), and estrogen receptor-binding effects (Vini et al., 2022). Eggerthellaceae is likely enriched in the high browse category due to the specific species of browse that were offered as opposed to the absolute quantity of leafy material. While many species contain ellagitannins (or ellagic acid), they are abundant in mulberry (*Morus* spp.), which is routinely offered to zoo-housed TKs (Roghan et al., 2024; Dierenfeld, Bezjian & Dabek, 2021). This result highlights the role of gut microbiota in enhancing bioavailability of absorbable metabolites by breaking down complex plant polyphenols (Pasinetti et al., 2018). However, it also illustrates the importance of browse species selection and the potential impacts on gut microbiome. Future studies exploring the impact of browse varieties and diversity on gut microbiota, host behavior, and health could offer valuable insights into how varying plant diets affect animal well-being. This research may enhance our understanding of foraging strategies, disease susceptibility, and the possibility of using dietary management as a targeted approach to improve animal health.

The high browse category is also associated with significant population decreases in *Turicibacter*, a genus that is common in the gastrointestinal tract of animals and is highly heritable (Goodrich et al., 2016; Loudon et al., 2022). These bacteria modify host bile acids and host metabolism, particularly lipid metabolism (Lynch et al., 2023). Generally, enriched *Turicibacter* is associated with inflammation, adipose tissue mass, and cancer in mice and humans (Zackular et al., 2013; Islam et al., 2021), however, there may be differential effects at the species and strain level (Lynch et al., 2023). The connection between diet and relative abundance of this taxon is unclear. Hao et al. (2021) found that moderately high soy fiber diets resulted in enriched abundance. Our findings suggest that high fiber diets are associated with a depleted abundance of *Turicibacter*. This may be an artifact of high browse diets having lower quantities of soy-based commercial-biscuits and not necessarily due to increased browse or overall fiber in the provided diets. Overall, the significant differences between browse categories in the weighted and unweighted UniFrac metrics combined with the relative abundance differences observed in the high browse category supports previous findings that diets high in browse promote gastrointestinal health in folivores (Greene et al., 2018; Plocek, Amato & Dunham, 2022).

## Conclusions

Like many other zoo-housed folivores, Matschie’s TKs are routinely fed diets that differ remarkably in macronutrient composition from those of their wild counterparts (Dierenfeld et al., 2020; Plocek & Dunham, 2024). Within the last decade, researchers investigating the gut microbiome have found diet to play a meaningful role in bacterial community makeup and structure across a multitude of species, and disturbances to these microbial communities are associated with decreased overall health (Chong et al., 2020; Clayton et al., 2016; Kohl, Skopec & Dearing, 2014; Wasimuddin et al., 2017; McKenzie et al., 2017). At present, the population of TKs in institutions accredited by the Association of Zoos and Aquariums is not self-sustaining, likely related to the presence of disease(s) of unknown etiology as well as obesity-related low overall reproductive success (Travis, Watson & Dabek, 2012; Dunham et al., 2022). Our study highlights significant variation in the dietary intake of zoo-housed TKs, with over-conditioned individuals consuming a greater number of calories, driven by increased CP and starch intake. The amount of leafy browse offered as part of the diet also significantly impacted the gut microbiome composition of this species, with higher browse offerings associated with enriching key microbial taxa. While additional investigations of the TK gut microbiome are warranted, including examinations of free-ranging individuals from Papua New Guinea, our study highlights the variation in diet offerings across zoos and reveals how this variation influences body condition, shapes gut microbial composition, and ultimately contributes to gastrointestinal health.

## Supplemental Information

10.7717/peerj.20875/supp-1 Supplemental Information 1 Effects of sex on dietary intake and pairwise comparisons of estimated marginal means between sex categories in North American zoo-housed Matschie’s tree kangaroos (*Dendrolagus matschiei*). Parentheses contain 95% confidence intervals. Shared superscripts within a row indicate that estimated marginal means were not significantly different. DM = dry matter; CP = crude protein; NDF = neutral detergent fiber; SC = soluble carbohydrates; CF = crude fat; df = degrees of freedom; df = degrees of freedom

10.7717/peerj.20875/supp-2 Supplemental Information 2 Effects of age on dietary intake in North American zoo-housed Matschie’s tree kangaroos (*Dendrolagus matschiei*). DM = dry matter; CP = crude protein; NDF = neutral detergent fiber; SC = soluble carbohydrates; CF = crude fat; df = degrees of freedom; df = degrees of freedom

10.7717/peerj.20875/supp-3 Supplemental Information 3 Supplementary Figures.

10.7717/peerj.20875/supp-4 Supplemental Information 4 Diet and animal condition raw data.

10.7717/peerj.20875/supp-5 Supplemental Information 5 Interactive hierarchical Krona chart illustrating the relative abundance of different bacterial taxa in the tree kangaroo gut microbiome.

## Additional Information and Declarations

### DNA Deposition

The following information was supplied regarding the deposition of DNA sequences:

The microbial sequence data are available at NIH NCBI BioProject: PRJNA1252074.

### Data Availability

The following information was supplied regarding data availability:

The data is available at Dryad: Koester, Diana; Plocek, Maura; Dierenfeld, Ellen et al. (2025). Data from: Variation in zoo diets, offerings of leafy browse, and body condition scores in Matschie’s tree kangaroos (*Dendrolagus matschiei*) and their effects on the composition of the gut microbiome [Dataset]. Dryad. https://doi.org/10.5061/dryad.mgqnk999g.
