# Supplementary material for: Variation in zoo diets, offerings of leafy browse, and body condition scores in Matschie’s tree kangaroos (Dendrolagus matschiei) and their associations with gut microbiome composition
Source: PeerJ. 2026 Feb 19;14:e20875. doi: 10.7717/peerj.20875 (PMC12925411; doi:10.7717/peerj.20875)
Supplement: Supplemental Information 1 — Parentheses contain 95% confidence intervals. Shared superscripts within a row indicate that estimated marginal means were not significantly different. DM = dry matter; CP = crude protein; NDF = neutral detergent fiber; SC = soluble carbohydrates; CF = crude fat; df = degrees of freedom; df = degrees of freedom [file peerj-14-20875-s001.docx]

Table S1. Effects of sex on dietary intake and pairwise comparisons of estimated marginal means between sex categories in North American zoo-housed Matschie’s tree kangaroos (*Dendrolagus matschiei*). Parentheses contain 95% confidence intervals. Shared superscripts within a row indicate that estimated marginal means were not significantly different.

| Parameter | F-value | df | P-value | Female | Male |
| --- | --- | --- | --- | --- | --- |
| DM g | 6.3 | 1, 21.9 | **0.02** | 113.7^a^  (91.2 – 136.1) | 150.5^b^  (129.6 – 171.3) |
| CP g | 8.1 | 1, 22.1 | **0.009** | 23.1^a^  (19.0 – 27.3) | 30.8^b^  (27.0 – 34.7) |
| NDF g | 6.0 | 1, 23.1 | **0.02** | 28.2^a^  (22.0 – 34.4) | 38.2 ^b^  (32.4 – 43.9) |
| Starch g | 2.5 | 1, 21.7 | 0.13 | 18.9^a^  (14.7 – 23.1) | 23.3 ^a^  (19.3 – 27.2) |
| SC g | 5.2 | 1, 23.0 | **0.03** | 14.1^a^  (11.1 – 17.2) | 18.7 ^b^  (15.8 – 21.6) |
| CF g | 6.2 | 1, 23.4 | **0.02** | 5.9^a^  (4.7 – 7.1) | 7.8 ^b^  (6.7 – 8.9) |
| Kcal | 6.5 | 1, 22.7 | **0.02** | 396.9^a^  (320.5 – 473.2) | 523.7 ^b^  (452.9 – 594.6) |

DM = dry matter; CP = crude protein; NDF = neutral detergent fiber; SC = soluble carbohydrates; CF = crude fat; df = degrees of freedom; df = degrees of freedom
