# Supplementary material for: Variation in zoo diets, offerings of leafy browse, and body condition scores in Matschie’s tree kangaroos (Dendrolagus matschiei) and their associations with gut microbiome composition
Source: PeerJ. 2026 Feb 19;14:e20875. doi: 10.7717/peerj.20875 (PMC12925411; doi:10.7717/peerj.20875)
Supplement: Supplemental Information 2 — DM = dry matter; CP = crude protein; NDF = neutral detergent fiber; SC = soluble carbohydrates; CF = crude fat; df = degrees of freedom; df = degrees of freedom [file peerj-14-20875-s002.docx]

Table S2. Effects of age on dietary intake in North American zoo-housed Matschie’s tree kangaroos (*Dendrolagus matschiei*).

| Parameter | F-value | df | P-value |
| --- | --- | --- | --- |
| DM g | 0.7 | 1, 21.9 | 0.43 |
| CP g | 2.5 | 1, 21.1 | 0.13 |
| NDF g | 1.2 | 1, 22.1 | 0.28 |
| Starch g | 0.2 | 1, 21.0 | 0.69 |
| SC g | 0.7 | 1, 22.9 | 0.40 |
| CF g | 0.6 | 1, 22.6 | 0.46 |
| Kcal | 0.6 | 1, 21.8 | 0.44 |

DM = dry matter; CP = crude protein; NDF = neutral detergent fiber; SC = soluble carbohydrates; CF = crude fat; df = degrees of freedom; df = degrees of freedom
