# Supplementary material for: Variation in zoo diets, offerings of leafy browse, and body condition scores in Matschie’s tree kangaroos (Dendrolagus matschiei) and their associations with gut microbiome composition
Source: PeerJ. 2026 Feb 19;14:e20875. doi: 10.7717/peerj.20875 (PMC12925411; doi:10.7717/peerj.20875)
Supplement: Supplemental Information 3 [file peerj-14-20875-s003.docx]

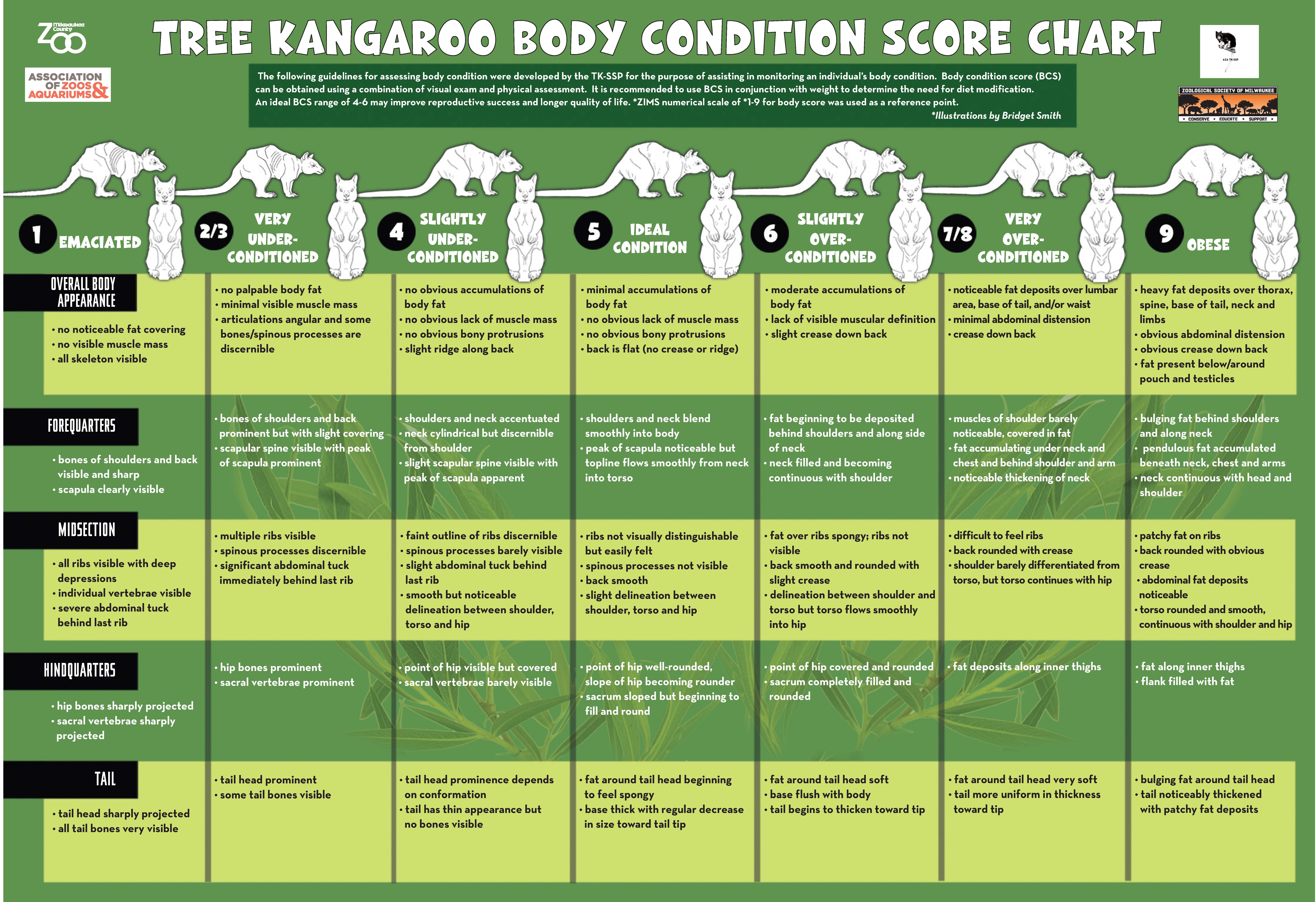
Fig Fig S1. Tree kangaroo body condition score chart.


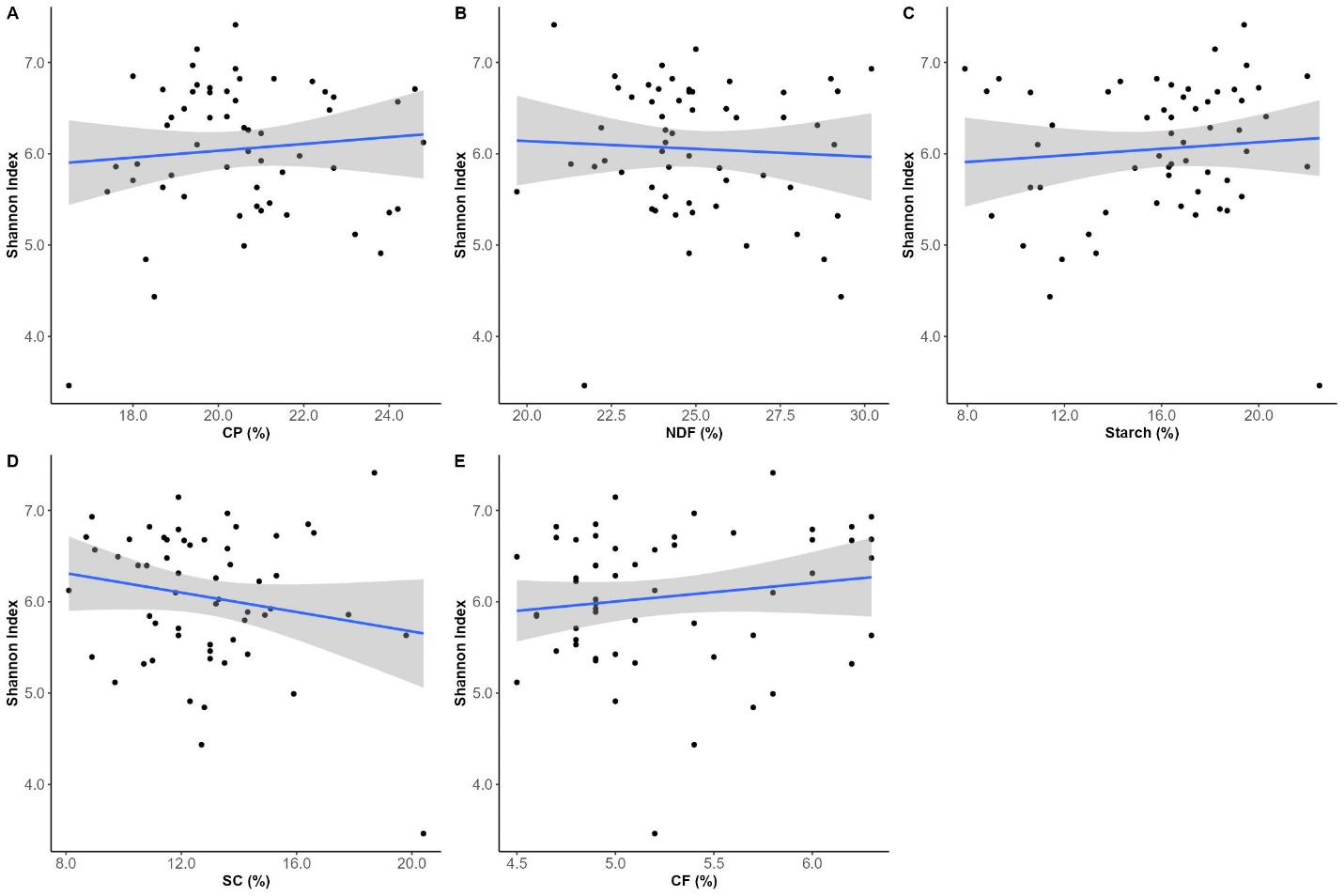


Figure S2. Effect of diet intake (measured as the relative proportions of dry matter consumed) on Shannon diversity.


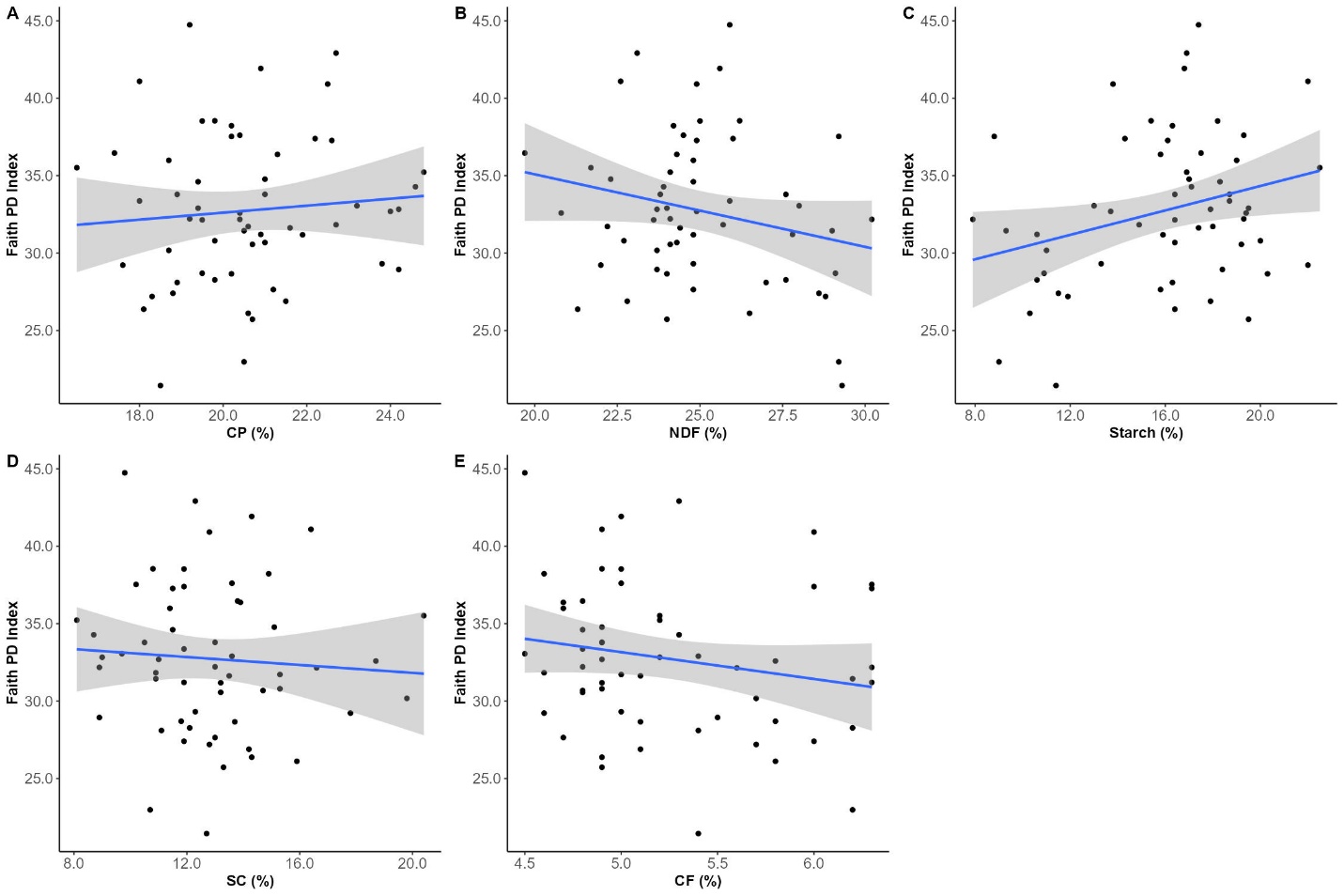


Figure S3. Effect of diet intake (measured as the relative proportions of dry matter consumed) on Faith’s phylogenetic diversity index.


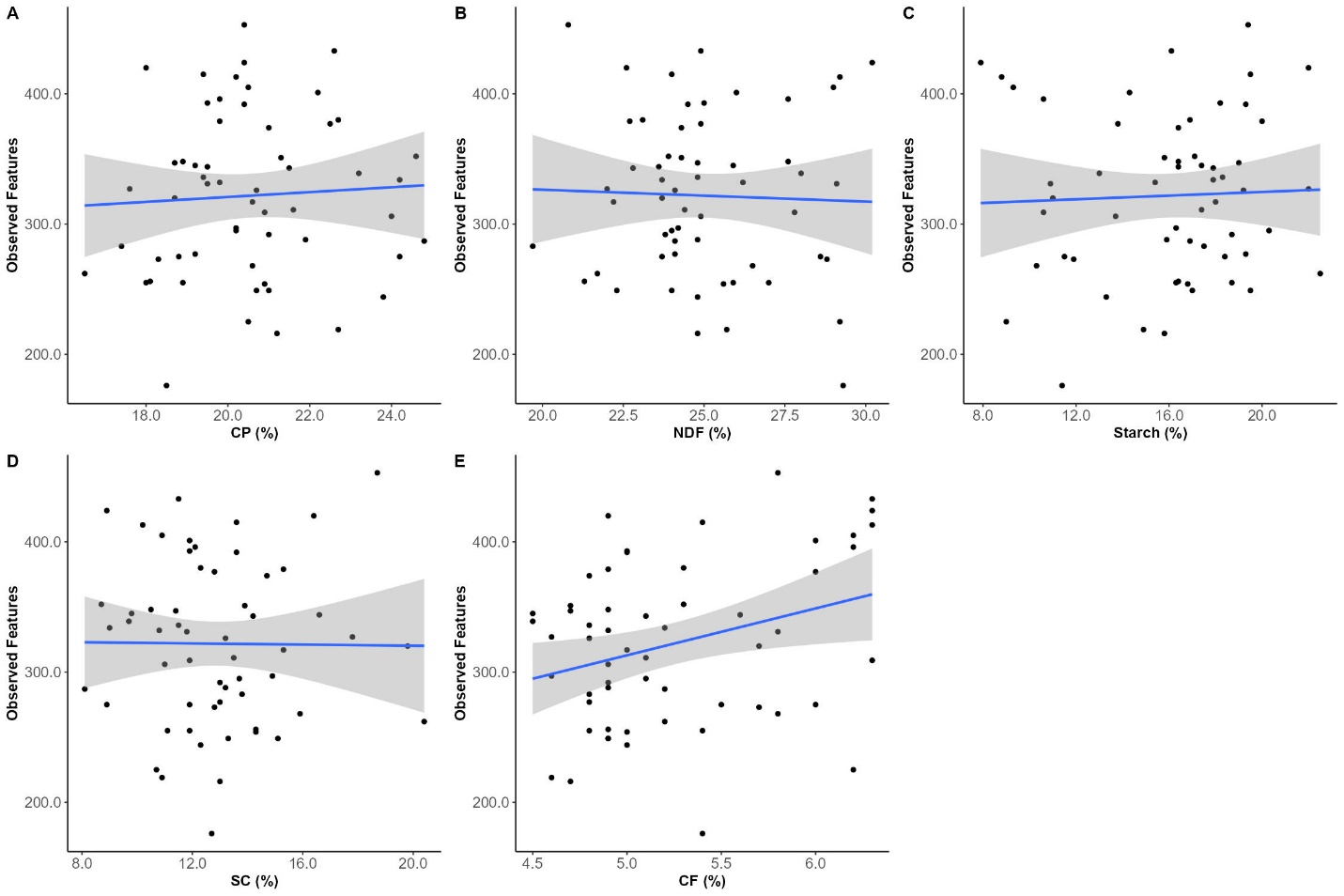


Figure S4. Effect of diet intake (measured as the relative proportions of dry matter consumed) on the number of observed features (i.e., amplicon sequence variants).


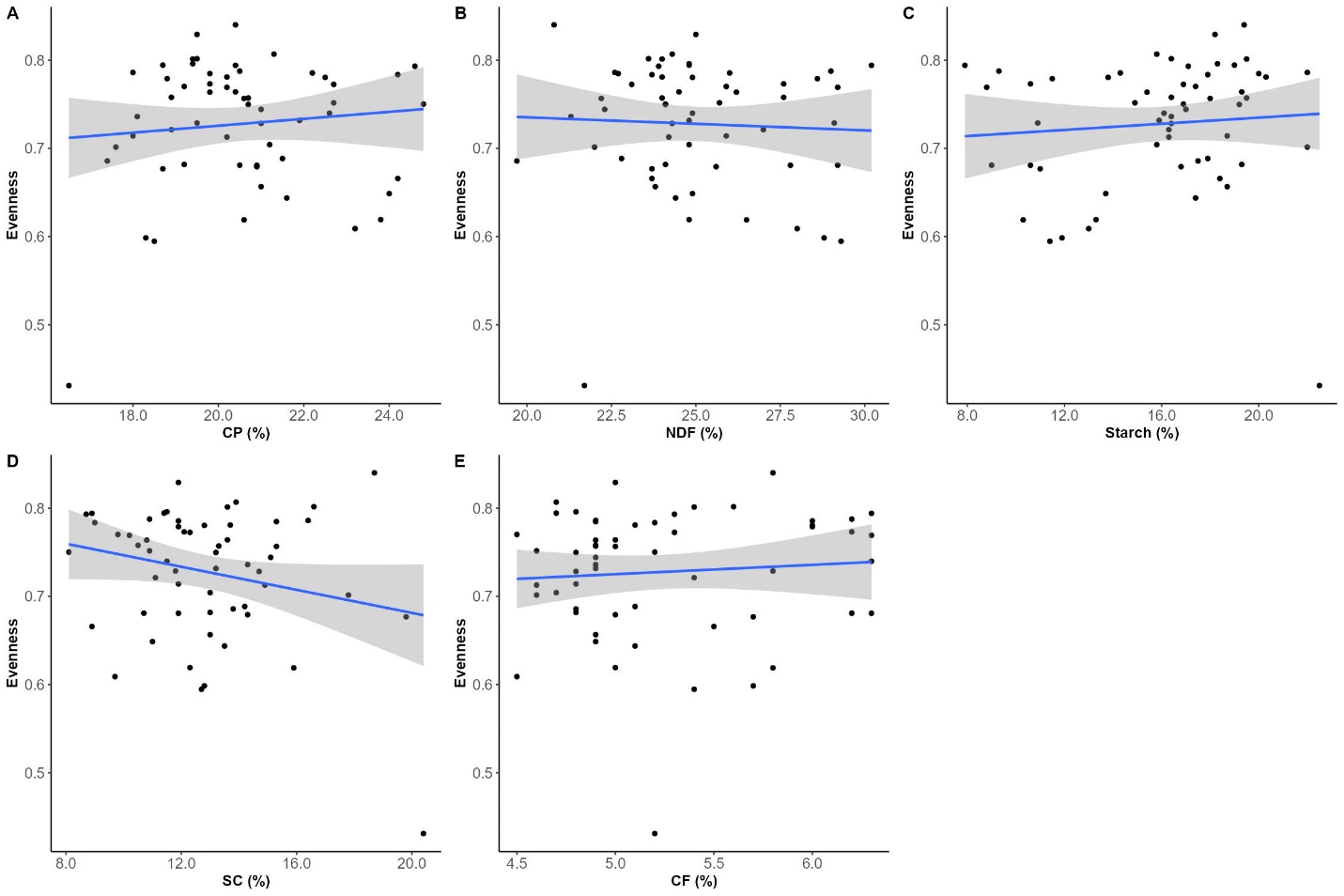


Figure S5. Effect of diet intake (measured as the relative proportions of dry matter consumed) on evenness.

Figure S6. Betadisper analysis comparing within group dispersion for browse category in unweighted (A) and weighted (B) dissimilarity matrices, and body condition score (BCS) in unweighted (C) and weighted (D) dissimilarity matrices.
