# Supplementary material for: Variation in zoo diets, offerings of leafy browse, and body condition scores in Matschie’s tree kangaroos (Dendrolagus matschiei) and their associations with gut microbiome composition
Source: PeerJ. 2026 Feb 19;14:e20875. doi: 10.7717/peerj.20875 (PMC12925411; doi:10.7717/peerj.20875)
Supplement: Supplemental Information 5 [file peerj-14-20875-s005.zip › Koester et al_Supplemental Material_TK_krona_ASV.html]

Javascript must be enabled to view this page.

magnitude
magnitudeUnassigned

Number.of.ASVs\_temp

10283

45

8

8

8

8

1

1

1

1

1

1

1

1

1

10

10

7

7

1

1

1

1

1

1

1

3

3

1

1

1

1

1

9

9

9

9

1

1

1

1

1

1

1

1

1

1

5

5

5

1

1

4

1

1

1

1

7

1

1

1

1

6

6

6

1

1

1

1

1

1

1

1

105

1

1

1

1

1

1

1

1

1

1

1

1

1

1

1

1

1

1

1

1

1

1

1

1

1

1

1

1

1

1

1

1

1

1

1

1

1

1

1

1

1

1

1

1

1

1

1

1

1

1

1

1

1

1

1

1

1

1

1

1

1

1

1

1

1

1

1

1

1

1

1

1

1

1

1

1

1

1

1

1

1

1

1

1

1

1

1

1

1

1

1

1

1

1

1

1

1

1

1

1

1

1

1

1

1

2

2

2

2

2

1

1

10131

1

1

1

12

12

12

12

1

1

1

1

1

1

1

1

1

1

1

1

1

14

14

14

14

1

1

1

1

1

1

1

1

1

1

1

1

1

1

1

1

1

1

1

1

1

1

1

1

1

1

1

1

1

1

1

1

1

1

1

1

1

1

1

1

1

1

1

1

1

160

73

44

34

1

1

1

1

1

1

1

1

1

1

1

1

1

1

1

1

1

1

1

1

1

1

1

1

1

1

1

1

1

1

1

1

1

1

10

1

1

1

1

1

1

1

1

1

1

2

2

1

1

1

1

1

1

1

1

1

24

24

1

1

1

1

1

1

1

1

1

1

1

1

1

1

1

1

1

1

1

1

1

1

1

1

4

4

2

1

1

1

1

1

57

9

9

1

1

1

1

1

1

1

1

1

20

20

1

1

1

1

1

1

1

1

1

1

1

1

1

1

1

1

1

1

1

1

28

24

1

1

1

1

1

1

1

1

1

1

1

1

1

1

1

1

1

1

1

1

1

1

1

1

3

1

1

1

1

1

26

26

26

1

1

1

1

1

1

1

1

1

1

1

1

1

1

1

1

1

1

1

1

1

1

1

1

1

1

1

1

1

2

2

1

1

1

1

1

1

1

1

1

1

1

1

1

1696

2

1

1

1

1

1

1

1

1

1

1

1

1

1

1

1

1691

1

1

1

1

1

1

1

1

1

1

1

1

1

1

1

1

1

77

2

1

1

4

1

1

1

1

1

9

1

1

1

1

1

1

1

1

1

1

4

1

1

1

1

53

1

1

1

1

1

1

1

1

1

1

1

1

1

1

1

1

1

1

1

1

1

1

1

1

1

1

1

1

1

1

1

1

1

1

1

1

1

1

1

1

1

1

1

1

1

1

1

1

1

1

1

1

1

2

1

1

1

1

1

1

1

1

1

1

1

1

1

1

1

1

1

1

1

1

1

1

1

1

1

1

1

1

1

1

1

1

26

1

1

5

1

1

1

1

1

2

1

1

17

1

1

1

1

1

1

1

1

1

1

1

1

1

1

1

1

1

1

1

1

1

1

1

1

1

1

1

1

1

1

1

1

1

1

1

1

1

1

1497

1

36

1

1

1

1

1

1

1

1

1

1

1

1

1

1

1

1

1

1

1

1

1

1

1

1

1

1

1

1

1

1

1

1

1

1

1

1

1

1

1

3

1

1

1

1

1

1

1

1

1

1

1

1

1

1

1

1

1

4

1

1

1

1

1

1

1

176

1

1

1

1

1

1

1

1

1

1

1

1

1

1

1

1

1

1

1

1

1

1

1

1

1

1

1

1

1

1

1

1

1

1

1

1

1

1

1

1

1

1

1

1

1

1

1

1

1

1

1

1

1

1

1

1

1

1

1

1

1

1

1

1

1

1

1

1

1

1

1

1

1

1

1

1

1

1

1

1

1

1

1

1

1

1

1

1

1

1

1

1

1

1

1

1

1

1

1

1

1

1

1

1

1

1

1

1

1

1

1

1

1

1

1

1

1

1

1

1

1

1

1

1

1

1

1

1

1

1

1

1

1

1

1

1

1

1

1

1

1

1

1

1

1

1

1

1

1

1

1

1

1

1

1

1

1

1

1

1

1

1

1

1

1

1

1

1

1

1

1

1

1

1

1

1

1

1

1

3

1

1

1

1

1

1

1

1

1

1

1

1

1

1

1

1

1

1

1

1

1

1

1

1

1

1

1

1

1

1

1

1

1

1

1

1

1

1

1

1

1

1

1

1

1

9

1

1

1

1

1

1

1

1

1

1

1

1

1

1

1

1

1

1

1

1

1

1

1

1

1

1

1

1

1

1

1

1

1

1

1

1

1

1

1

1

22

1

1

1

1

1

1

1

1

1

1

1

1

1

1

1

1

1

1

1

1

1

1

1

1

1

1

1

1

1

1

1

1

1

1

1

1

1

1

1

1

1

1

1

1

1

1

1

1

1

1

410

1

1

1

1

1

1

1

1

1

1

1

1

1

1

1

1

1

1

1

1

1

1

1

1

1

1

1

1

1

1

1

1

1

1

1

1

1

1

1

1

1

1

1

1

1

1

1

1

1

1

1

1

1

1

1

1

1

1

1

1

1

1

1

1

1

1

1

1

1

1

1

1

1

1

1

1

1

1

1

1

1

1

1

1

1

1

1

1

1

1

1

1

1

1

1

1

1

1

1

1

1

1

1

1

1

1

1

1

1

1

1

1

1

1

1

1

1

1

1

1

1

1

1

1

1

1

1

1

1

1

1

1

1

1

1

1

1

1

1

1

1

1

1

1

1

1

1

1

1

1

1

1

1

1

1

1

1

1

1

1

1

1

1

1

1

1

1

1

1

1

1

1

1

1

1

1

1

1

1

1

1

1

1

1

1

1

1

1

1

1

1

1

1

1

1

1

1

1

1

1

1

1

1

1

1

1

1

1

1

1

1

1

1

1

1

1

1

1

1

1

1

1

1

1

1

1

1

1

1

1

1

1

1

1

1

1

1

1

1

1

1

1

1

1

1

1

1

1

1

1

1

1

1

1

1

1

1

1

1

1

1

1

1

1

1

1

1

1

1

1

1

1

1

1

1

1

1

1

1

1

1

1

1

1

1

1

1

1

1

1

1

1

1

1

1

1

1

1

1

1

1

1

1

1

1

1

1

1

1

1

1

1

1

1

1

1

1

1

1

1

1

1

1

1

1

1

1

1

1

1

1

1

1

1

1

1

1

1

1

1

1

1

1

1

1

1

1

1

1

1

1

1

1

1

1

1

1

1

1

1

1

1

1

1

1

1

1

1

1

1

1

1

1

1

1

1

1

1

1

1

1

1

1

1

1

1

1

1

1

1

1

1

1

1

1

1

1

1

1

1

1

1

1

1

1

1

1

1

1

1

2

1

1

1

1

1

223

1

1

1

1

1

1

1

1

1

1

1

1

1

1

1

1

1

1

1

1

1

1

1

1

1

1

1

1

1

1

1

1

1

1

1

1

1

1

1

1

1

1

1

1

1

1

1

1

1

1

1

1

1

1

1

1

1

1

1

1

1

1

1

1

1

1

1

1

1

1

1

1

1

1

1

1

1

1

1

1

1

1

1

1

1

1

1

1

1

1

1

1

1

1

1

1

1

1

1

1

1

1

1

1

1

1

1

1

1

1

1

1

1

1

1

1

1

1

1

1

1

1

1

1

1

1

1

1

1

1

1

1

1

1

1

1

1

1

1

1

1

1

1

1

1

1

1

1

1

1

1

1

1

1

1

1

1

1

1

1

1

1

1

1

1

1

1

1

1

1

1

1

1

1

1

1

1

1

1

1

1

1

1

1

1

1

1

1

1

1

1

1

1

1

1

1

1

1

1

1

1

1

1

1

1

1

1

1

1

1

1

1

1

1

1

1

1

1

1

1

1

1

1

1

1

1

1

1

1

1

1

1

1

1

1

1

1

1

1

1

1

1

1

1

1

1

1

1

1

1

1

1

1

1

1

1

1

1

22

1

1

1

1

1

1

1

1

1

1

1

1

1

1

1

1

1

1

1

1

1

1

1

1

1

1

1

1

1

1

1

1

1

1

1

1

1

45

1

1

1

1

1

1

1

1

1

1

1

1

1

1

1

1

1

1

1

1

1

1

1

1

1

1

1

1

1

1

1

1

1

1

1

1

1

1

1

1

1

1

1

1

1

1

1

1

1

1

1

1

1

1

1

1

1

1

1

1

1

1

1

1

1

265

1

1

1

1

1

1

1

1

1

1

1

1

1

1

1

1

1

1

1

1

1

1

1

1

1

1

1

1

1

1

1

1

1

1

1

1

1

1

1

1

1

1

1

1

1

1

1

1

1

1

1

1

1

1

1

1

1

1

1

1

1

1

1

1

1

1

1

1

1

1

1

1

1

1

1

1

1

1

1

1

1

1

1

1

1

1

1

1

1

1

1

1

1

1

1

1

1

1

1

1

1

1

1

1

1

1

1

1

1

1

1

1

1

1

1

1

1

1

1

1

1

1

1

1

1

1

1

1

1

1

1

1

1

1

1

1

1

1

1

1

1

1

1

1

1

1

1

1

1

1

1

1

1

1

1

1

1

1

1

1

1

1

1

1

1

1

1

1

1

1

1

1

1

1

1

1

1

1

1

1

1

1

1

1

1

1

1

1

1

1

1

1

1

1

1

1

1

1

1

1

1

1

1

1

1

1

1

1

1

1

1

1

1

1

1

1

1

1

1

1

1

1

1

1

1

1

1

1

1

1

1

1

1

1

1

1

1

1

1

1

1

1

1

1

1

1

1

1

1

1

1

1

1

1

1

1

1

1

1

1

1

1

1

1

1

1

1

1

21

1

1

1

1

1

1

1

1

1

1

1

1

1

1

1

1

1

1

1

1

1

1

1

1

1

1

1

6

1

1

1

1

1

1

3

1

1

1

1

1

1

1

1

1

1

1

1

1

1

1

1

1

1

1

1

1

1

1

1

1

1

1

1

1

1

1

1

10

1

1

1

1

1

1

1

1

1

1

1

1

1

1

1

1

1

1

1

1

1

1

1

1

1

1

4

2

1

1

2

1

1

1

1

4

1

1

1

2

1

1

1

1

1

1

2

2

2

2

1

1

2

2

2

2

1

1

1

1

1

1

1

1

1

1

1

1

1

1

33

1

1

1

1

4

4

4

1

1

1

1

27

1

1

1

26

26

1

1

1

1

1

1

1

1

1

1

1

1

1

1

1

1

1

1

1

1

1

1

1

1

1

1

1

1

1

1

1

1

1

1

1

1

69

4

4

2

1

1

2

1

1

18

18

18

1

1

1

1

1

1

1

1

1

1

1

1

1

1

1

1

1

1

1

1

1

1

45

45

45

1

1

1

1

1

1

1

1

1

1

1

1

1

1

1

1

1

1

1

1

1

1

1

1

1

1

1

1

1

1

1

1

1

1

1

1

1

1

1

1

1

1

1

1

1

1

1

1

1

1

1

1

1

1

1

1

7043

1

1

1

1

1

1

1

1

1

1

1

1

1

1

1

1

1

1

1

1

1

1

1

1

1

1

63

27

27

1

1

1

1

1

1

1

1

1

1

1

1

1

1

1

1

1

1

1

1

1

1

1

1

1

1

1

36

1

1

17

1

1

1

1

1

1

1

1

1

1

1

1

1

1

1

1

1

1

17

1

1

1

1

1

1

1

1

1

1

1

1

1

1

1

1

1

1

1

6279

1

1

1

1

1

1

1

1

1

1

1

1

1

1

1

1

1

1

1

1

1

1

1

1

1

1

1

1

1

1

1

1

1

1

1

1

1

1

1

1

1

1

1

1

1

1

1

1

1

1

1

1

1

1

1

1

1

1

1

1

1

1

1

1

1

1

1

1

1

1

1

1

1

1

1

1

1

153

153

1

1

1

1

1

1

1

1

1

1

1

1

1

1

1

1

1

1

1

1

1

1

1

1

1

1

1

1

1

1

1

1

1

1

1

1

1

1

1

1

1

1

1

1

1

1

1

1

1

1

1

1

1

1

1

1

1

1

1

1

1

1

1

1

1

1

1

1

1

1

1

1

1

1

1

1

1

1

1

1

1

1

1

1

1

1

1

1

1

1

1

1

1

1

1

1

1

1

1

1

1

1

1

1

1

1

1

1

1

1

1

1

1

1

1

1

1

1

1

1

1

1

1

1

1

1

1

1

1

1

1

1

1

1

1

1

1

1

1

1

1

1

1

1

1

1

1

1

1

1

1

1

1

1

1

1

1

1

1

1

1

1

1

1

1

1

1

1

1

1

1

1

1

1

1

1

1

1

1

1

1

1

1

1

1

1

1

1

1

1

1

1

1

1

1

1

1

1

1

1

1

1

1

1

1

1

158

1

1

2

1

1

111

1

1

1

1

1

1

1

1

1

1

1

1

1

1

1

1

1

1

1

1

1

1

1

1

1

1

1

1

1

1

1

1

1

1

1

1

1

1

1

1

1

1

1

1

1

1

1

1

1

1

1

1

1

1

1

1

1

1

1

1

1

1

1

1

1

1

1

1

1

1

1

1

1

1

1

1

1

1

1

1

1

1

1

1

1

1

1

1

1

1

1

1

1

1

1

1

1

1

1

1

1

1

1

1

1

1

1

1

1

1

1

7

1

1

1

1

1

1

1

1

1

1

30

1

1

1

1

1

1

1

1

1

1

1

1

1

1

1

1

1

1

1

1

1

1

1

1

1

1

1

1

1

1

1

1

1

1

1

1

1

1

1

1

1

1

1

1

1

1

1

1

1

1

1

1

1

1

1

1

1

1

1

1

1

1

1

1

1

1

1

1

1

1

1

1

1

1

1

1

1

1

1

1

1

1

1

1

1

1

1

1

1

1

1

1

1

1

1

1

1

1

1

1

1

1

1

1

1

1

1

1

1

1

1

1

1

1

1

1

1

1

1

1

1

1

1

1

1

1

1

1

1

1

41

41

1

1

1

1

1

1

1

1

1

1

1

1

1

1

1

1

1

1

1

1

1

1

1

1

1

1

1

1

1

1

1

1

1

1

1

1

1

1

1

1

1

1

1

1

1

1

1

1

1

1

1

1

1

1

1

1

1

1

1

1

1

1

1

1

1

1

1

1

1

1

1

1

1

1

1

1

1

1

1

1

1

1

1

1

1

1

1

1

1

1

1

1

1

1

1

1

1

1

1

1

1

1

1

1

1

1

1

1

1

1

1

1

1

1

1

1

1

1

1

1

1

1

1

1

1

1

1

1

1

1

1

1

1

1

1

1

1

1

1

1

1

1

1

1

1

1

1

1

1

1

1

1

1

1

1

1

1

1

1

1

1

1

1

1

1

1

1

1

1

1

1

68

68

1

1

1

1

1

1

1

1

1

1

1

1

1

1

1

1

1

1

1

1

1

1

1

1

1

1

1

1

1

1

1

1

1

1

1

1

1

1

1

1

1

1

1

1

1

1

1

1

1

1

1

1

1

1

1

1

1

1

1

1

1

1

1

1

1

1

1

1

1

1

1

1

1

1

1

1

1

1

1

1

1

1

1

1

1

1

1

1

1

1

1

1

1

1

1

1

1

1

1

1

385

385

1

1

1

1

1

1

1

1

1

1

1

1

1

1

1

1

1

1

1

1

1

1

1

1

1

1

1

1

1

1

1

1

1

1

1

1

1

1

1

1

1

1

1

1

1

1

1

1

1

1

1

1

1

1

1

1

1

1

1

1

1

1

1

1

1

1

1

1

1

1

1

1

1

1

1

1

1

1

1

1

1

1

1

1

1

1

1

1

1

1

1

1

1

1

1

1

1

1

1

1

1

1

1

1

1

1

1

1

1

1

1

1

1

1

1

1

1

1

1

1

1

1

1

1

1

1

1

1

1

1

1

1

1

1

1

1

1

1

1

1

1

1

1

1

1

1

1

1

1

1

1

1

1

1

1

1

1

1

1

1

1

1

1

1

1

1

1

1

1

1

1

1

1

1

1

1

1

1

1

1

1

1

1

1

1

1

1

1

1

1

1

1

1

1

1

1

1

1

1

1

1

1

1

1

1

1

1

1

1

1

1

1

1

1

1

1

1

1

1

1

1

1

1

1

1

1

1

1

1

1

1

1

1

1

1

1

1

1

1

1

1

1

1

1

1

1

1

1

1

1

1

1

1

1

1

1

1

1

1

1

1

1

1

1

1

1

1

1

1

1

1

1

1

1

1

1

1

1

1

1

1

1

1

1

1

1

1

1

1

1

1

1

1

1

1

1

1

1

1

1

1

1

1

1

1

1

1

1

1

1

1

1

1

1

1

1

1

1

1

1

1

1

1

1

1

1

1

1

1

1

1

1

1

1

1

1

1

1

1

1

1

1

1

1

1

1

1

1

1

1

1

1

1

1

1

1

1

1

1

1

1

1

1

1

1

1

1

1

1

1

1

1

1

1

1

1

1

1

1

1

1

1

1

1

1

1

1

1

1

1

1

1

1

1

1

1

1

1

1

1

1

1

1

1

1

1

1

1

1

1

1

1

1

1

1

1

1

1

1

1

1

1

1

1

1

1

41

40

1

1

1

1

1

1

1

1

1

1

1

1

1

1

1

1

1

1

1

1

1

1

1

1

1

1

1

1

1

1

1

1

1

1

1

1

1

1

1

1

1

1

1

1

1

1

1

1

1

1

1

1

1

1

1

1

1

1

1

1

1

1

1

1

1

1

1

1

1

1

1

1

1

1

1

1

1

1

1

1

1

1

1

1

1

1

1

1

1

1

1

1

1

1

1

1

1

1

1

1

1

1

1

1

1

1

1

1

1

1

1

1

1

1

1

11

1

3

1

1

1

7

1

1

1

1

1

1

1

1

1

1

1

1

1

1

1

2294

1

1

1

1

7

1

1

1

1

1

1

1

1

1

1

1

1

1

1

1

1

1

1

1

1

1

1

1

1

1

1

1

1

1

1

1

1

1

1

1

1

1

1

1

1

1

1

11

1

1

1

1

1

1

1

1

1

1

1

1

1

1

1

1

1

1

1

1

1

1

1

1

1

1

1

1

1

1

1

1

1

1

1

1

1

1

1

1

1

1

1

2

1

1

1

1

1

1

1

1

1

1

1

1

1

1

1

1

1

1

1

1

1

1

1

1

1

1

1

1

1

1

1

1

1

1

1

1

1

1

1

1

291

1

1

1

1

1

1

1

1

1

1

1

1

1

1

1

1

1

1

1

1

1

1

1

1

1

1

1

1

1

1

1

1

1

1

1

1

1

1

1

1

1

1

1

1

1

1

1

1

1

1

1

1

1

1

1

1

1

1

1

1

1

1

1

1

1

1

1

1

1

1

1

1

1

1

1

1

1

1

1

1

1

1

1

1

1

1

1

1

1

1

1

1

1

1

1

1

1

1

1

1

1

1

1

1

1

1

1

1

1

1

1

1

1

1

1

1

1

1

1

1

1

1

1

1

1

1

1

1

1

1

1

1

1

1

1

1

1

1

1

1

1

1

1

1

1

1

1

1

1

1

1

1

1

1

1

1

1

1

1

1

1

1

1

1

1

1

1

1

1

1

1

1

1

1

1

1

1

1

1

1

1

1

1

1

1

1

1

1

1

1

1

1

1

1

1

1

1

1

1

1

1

1

1

1

1

1

1

1

1

1

1

1

1

1

1

1

1

1

1

1

1

1

1

1

1

1

1

1

1

1

1

1

1

1

1

1

1

1

1

1

1

1

1

1

1

1

1

1

1

1

1

1

1

1

1

1

1

1

1

1

1

1

1

1

1

1

1

1

1

1

1

1

1

1

1

1

1

1

1

1

1

1

1

1

1

1

1

1

1

1

1

1

1

1

1

1

1

1

1

1

1

1

1

1

1

1

1

1

1

1

1

1

1

1

1

1

1

1

1

1

1

1

1

1

1

1

1

1

1

1

1

378

1

1

1

1

1

1

1

1

1

1

1

1

1

1

1

1

1

1

1

1

1

1

1

1

1

1

1

1

1

1

1

1

1

1

1

1

1

1

1

1

1

1

1

1

1

1

1

1

1

1

1

1

1

1

1

1

1

1

1

1

1

1

1

1

1

1

1

1

1

1

1

1

1

1

1

1

1

1

1

1

1

1

1

1

1

1

1

1

1

1

1

1

1

1

1

1

1

1

1

1

1

1

1

1

1

1

1

1

1

1

1

1

1

1

1

1

1

1

1

1

1

1

1

1

1

1

1

1

1

1

1

1

1

1

1

1

1

1

1

1

1

1

1

1

1

1

1

1

1

1

1

1

1

1

1

1

1

1

1

1

1

1

1

1

1

1

1

1

1

1

1

1

1

1

1

1

1

1

1

1

1

1

1

1

1

1

1

1

1

1

1

1

1

1

1

1

1

1

1

1

1

1

1

1

1

1

1

1

1

1

1

1

1

1

1

1

1

1

1

1

1

1

1

1

1

1

1

1

1

1

1

1

1

1

1

1

1

1

1

1

1

1

1

1

1

1

1

1

1

1

1

1

1

1

1

1

1

1

1

1

1

1

1

1

1

1

1

1

1

1

1

1

1

1

1

1

1

1

1

1

1

1

1

1

1

1

1

1

1

1

1

1

1

1

1

1

1

1

1

1

1

1

1

1

1

1

1

1

1

1

1

1

1

1

1

1

1

1

1

1

1

1

1

1

1

1

1

1

1

1

1

1

1

1

1

1

1

1

1

1

1

1

1

1

1

1

1

1

1

1

1

1

1

1

1

1

1

1

1

1

1

1

1

1

1

1

1

1

1

1

1

1

1

1

1

1

1

1

1

1

1

1

1

1

1

1

1

890

1

1

1

1

1

1

1

1

1

1

1

1

1

1

1

1

1

1

1

1

1

1

1

1

1

1

1

1

1

1

1

1

1

1

1

1

1

1

1

1

1

1

1

1

1

1

1

1

1

1

1

1

1

1

1

1

1

1

1

1

1

1

1

1

1

1

1

1

1

1

1

1

1

1

1

1

1

1

1

1

1

1

1

1

1

1

1

1

1

1

1

1

1

1

1

1

1

1

1

1

1

1

1

1

1

1

1

1

1

1

1

1

1

1

1

1

1

1

1

1

1

1

1

1

1

1

1

1

1

1

1

1

1

1

1

1

1

1

1

1

1

1

1

1

1

1

1

1

1

1

1

1

1

1

1

1

1

1

1

1

1

1

1

1

1

1

1

1

1

1

1

1

1

1

1

1

1

1

1

1

1

1

1

1

1

1

1

1

1

1

1

1

1

1

1

1

1

1

1

1

1

1

1

1

1

1

1

1

1

1

1

1

1

1

1

1

1

1

1

1

1

1

1

1

1

1

1

1

1

1

1

1

1

1

1

1

1

1

1

1

1

1

1

1

1

1

1

1

1

1

1

1

1

1

1

1

1

1

1

1

1

1

1

1

1

1

1

1

1

1

1

1

1

1

1

1

1

1

1

1

1

1

1

1

1

1

1

1

1

1

1

1

1

1

1

1

1

1

1

1

1

1

1

1

1

1

1

1

1

1

1

1

1

1

1

1

1

1

1

1

1

1

1

1

1

1

1

1

1

1

1

1

1

1

1

1

1

1

1

1

1

1

1

1

1

1

1

1

1

1

1

1

1

1

1

1

1

1

1

1

1

1

1

1

1

1

1

1

1

1

1

1

1

1

1

1

1

1

1

1

1

1

1

1

1

1

1

1

1

1

1

1

1

1

1

1

1

1

1

1

1

1

1

1

1

1

1

1

1

1

1

1

1

1

1

1

1

1

1

1

1

1

1

1

1

1

1

1

1

1

1

1

1

1

1

1

1

1

1

1

1

1

1

1

1

1

1

1

1

1

1

1

1

1

1

1

1

1

1

1

1

1

1

1

1

1

1

1

1

1

1

1

1

1

1

1

1

1

1

1

1

1

1

1

1

1

1

1

1

1

1

1

1

1

1

1

1

1

1

1

1

1

1

1

1

1

1

1

1

1

1

1

1

1

1

1

1

1

1

1

1

1

1

1

1

1

1

1

1

1

1

1

1

1

1

1

1

1

1

1

1

1

1

1

1

1

1

1

1

1

1

1

1

1

1

1

1

1

1

1

1

1

1

1

1

1

1

1

1

1

1

1

1

1

1

1

1

1

1

1

1

1

1

1

1

1

1

1

1

1

1

1

1

1

1

1

1

1

1

1

1

1

1

1

1

1

1

1

1

1

1

1

1

1

1

1

1

1

1

1

1

1

1

1

1

1

1

1

1

1

1

1

1

1

1

1

1

1

1

1

1

1

1

1

1

1

1

1

1

1

1

1

1

1

1

1

1

1

1

1

1

1

1

1

1

1

1

1

1

1

1

1

1

1

1

1

1

1

1

1

1

1

1

1

1

1

1

1

1

1

1

1

1

1

1

1

1

1

1

1

1

1

1

1

1

1

1

1

1

1

1

1

1

1

1

1

1

1

1

1

1

1

1

1

1

1

1

1

1

1

1

1

1

1

1

1

1

1

1

1

1

1

1

1

1

1

1

1

1

1

1

1

1

1

1

1

1

1

1

1

1

1

1

1

1

1

1

1

1

1

1

1

1

1

1

1

1

1

1

1

1

1

1

1

1

1

1

1

1

1

1

1

1

1

1

1

1

1

1

1

1

1

1

1

1

1

1

1

1

1

1

1

1

1

1

1

1

1

1

1

1

1

1

1

1

1

1

1

1

1

1

1

1

1

1

1

1

1

1

1

1

1

1

1

1

1

1

1

1

1

1

1

1

1

1

1

1

1

1

1

1

1

1

1

1

1

1

1

1

1

1

1

1

1

1

1

1

1

1

1

1

1

1

1

1

1

1

1

1

1

1

1

1

1

1

1

1

1

1

1

1

1

1

1

1

1

1

1

1

1

1

1

1

1

60

1

1

1

1

1

1

1

1

1

1

1

1

1

1

1

1

1

1

1

1

1

1

1

1

1

1

1

1

1

1

1

1

1

1

1

1

1

1

1

1

1

1

1

1

1

1

1

1

1

1

1

1

1

1

1

1

1

1

1

1

1

1

1

1

1

1

1

1

1

1

1

1

1

1

1

1

1

1

1

1

1

1

1

1

1

1

1

1

1

1

1

1

442

1

1

1

1

1

1

1

1

1

1

1

1

1

1

1

1

1

1

1

1

1

1

1

1

1

1

1

1

1

1

1

1

1

1

1

1

1

1

1

1

1

1

1

1

1

1

1

1

1

1

1

1

1

1

1

1

1

1

1

1

1

1

1

1

1

1

1

1

1

1

1

1

1

1

1

1

1

1

1

1

1

1

1

1

1

1

1

1

1

1

1

1

1

1

1

1

1

1

1

1

1

1

1

1

1

1

1

1

1

1

1

1

1

1

1

1

1

1

1

1

1

1

1

1

1

1

1

1

1

1

1

1

1

1

1

1

1

1

1

1

1

1

1

1

1

1

1

1

1

1

1

1

1

1

1

1

1

1

1

1

1

1

1

1

1

1

1

1

1

1

1

1

1

1

1

1

1

1

1

1

1

1

1

1

1

1

1

1

1

1

1

1

1

1

1

1

1

1

1

1

1

1

1

1

1

1

1

1

1

1

1

1

1

1

1

1

1

1

1

1

1

1

1

1

1

1

1

1

1

1

1

1

1

1

1

1

1

1

1

1

1

1

1

1

1

1

1

1

1

1

1

1

1

1

1

1

1

1

1

1

1

1

1

1

1

1

1

1

1

1

1

1

1

1

1

1

1

1

1

1

1

1

1

1

1

1

1

1

1

1

1

1

1

1

1

1

1

1

1

1

1

1

1

1

1

1

1

1

1

1

1

1

1

1

1

1

1

1

1

1

1

1

1

1

1

1

1

1

1

1

1

1

1

1

1

1

1

1

1

1

1

1

1

1

1

1

1

1

1

1

1

1

1

1

1

1

1

1

1

1

1

1

1

1

1

1

1

1

1

1

1

1

1

1

1

1

1

1

1

1

1

1

1

1

1

1

1

1

1

1

1

1

1

1

1

1

1

1

1

1

1

1

1

1

1

1

1

1

1

1

1

1

1

1

1

1

1

1

1

1

1

1

1

1

1

1

1

1

1

1

1

1

1

1

1

1

1

1

1

1

1

1

1

1

1

1

1

1

1

1

1

1

1

1

1

1

1

1

1

1

1

1

1

1

1

1

1

1

1

1

1

1

1

1

1

1

1

1

1

1

1

1

1

1

1

1

1

1

1

1

1

1

1

1

1

1

1

1

1

1

1

1

1

1

1

1

1

1

1

1

1

1

1

27

1

1

25

1

1

1

1

1

1

1

1

1

1

1

1

1

1

1

1

1

1

1

1

1

1

1

1

1

1

1

1

1

1

1

1

1

1

1

1

1

1

1

1

1

2078

1

1

1

1

1

1

1

1

1

1

1

1

1

1

1

1

2054

1

1

1

1

1

1

1

1

1

1

1

1

1

1

1

1

1

1

1

1

1

1

1

1

1

1

1

1

1

1

1

1

1

1

1

1

1

1

1

1

1

1

1

1

1

1

1

1

1

1

1

1

1

1

1

1

1

1

1

1

1

1

1

1

1

1

1

1

1

1

1

1

1

1

1

1

1

1

1

1

1

1

1

1

1

1

1

1

1

1

1

1

1

1

1

1

1

1

1

1

1

1

1

1

1

1

1

1

1

1

1

1

1

1

1

1

1

1

1

1

1

1

1

1

1

1

1

1

1

1

1

1

1

1

1

1

1

1

1

1

1

1

1

1

1

1

1

1

1

1

1

1

1

1

1

1

1

1

1

1

1

1

1

1

1

1

1

1

1

1

1

1

1

1

1

1

1

1

1

1

1

1

1

1

1

1

1

1

1

1

1

1

1

1

1

1

1

1

1

1

1

1

1

1

1

1

1

1

1

1

1

1

1

1

1

1

1

1

1

1

1

1

1

1

1

1

1

1

1

1

1

1

1

1

1

1

1

1

1

1

1

1

1

1

1

1

1

1

1

1

1

1

1

1

1

1

1

1

1

1

1

1

1

1

1

1

1

1

1

1

1

1

1

1

1

1

1

1

1

1

1

1

1

1

1

1

1

1

1

1

1

1

1

1

1

1

1

1

1

1

1

1

1

1

1

1

1

1

1

1

1

1

1

1

1

1

1

1

1

1

1

1

1

1

1

1

1

1

1

1

1

1

1

1

1

1

1

1

1

1

1

1

1

1

1

1

1

1

1

1

1

1

1

1

1

1

1

1

1

1

1

1

1

1

1

1

1

1

1

1

1

1

1

1

1

1

1

1

1

1

1

1

1

1

1

1

1

1

1

1

1

1

1

1

1

1

1

1

1

1

1

1

1

1

1

1

1

1

1

1

1

1

1

1

1

1

1

1

1

1

1

1

1

1

1

1

1

1

1

1

1

1

1

1

1

1

1

1

1

1

1

1

1

1

1

1

1

1

1

1

1

1

1

1

1

1

1

1

1

1

1

1

1

1

1

1

1

1

1

1

1

1

1

1

1

1

1

1

1

1

1

1

1

1

1

1

1

1

1

1

1

1

1

1

1

1

1

1

1

1

1

1

1

1

1

1

1

1

1

1

1

1

1

1

1

1

1

1

1

1

1

1

1

1

1

1

1

1

1

1

1

1

1

1

1

1

1

1

1

1

1

1

1

1

1

1

1

1

1

1

1

1

1

1

1

1

1

1

1

1

1

1

1

1

1

1

1

1

1

1

1

1

1

1

1

1

1

1

1

1

1

1

1

1

1

1

1

1

1

1

1

1

1

1

1

1

1

1

1

1

1

1

1

1

1

1

1

1

1

1

1

1

1

1

1

1

1

1

1

1

1

1

1

1

1

1

1

1

1

1

1

1

1

1

1

1

1

1

1

1

1

1

1

1

1

1

1

1

1

1

1

1

1

1

1

1

1

1

1

1

1

1

1

1

1

1

1

1

1

1

1

1

1

1

1

1

1

1

1

1

1

1

1

1

1

1

1

1

1

1

1

1

1

1

1

1

1

1

1

1

1

1

1

1

1

1

1

1

1

1

1

1

1

1

1

1

1

1

1

1

1

1

1

1

1

1

1

1

1

1

1

1

1

1

1

1

1

1

1

1

1

1

1

1

1

1

1

1

1

1

1

1

1

1

1

1

1

1

1

1

1

1

1

1

1

1

1

1

1

1

1

1

1

1

1

1

1

1

1

1

1

1

1

1

1

1

1

1

1

1

1

1

1

1

1

1

1

1

1

1

1

1

1

1

1

1

1

1

1

1

1

1

1

1

1

1

1

1

1

1

1

1

1

1

1

1

1

1

1

1

1

1

1

1

1

1

1

1

1

1

1

1

1

1

1

1

1

1

1

1

1

1

1

1

1

1

1

1

1

1

1

1

1

1

1

1

1

1

1

1

1

1

1

1

1

1

1

1

1

1

1

1

1

1

1

1

1

1

1

1

1

1

1

1

1

1

1

1

1

1

1

1

1

1

1

1

1

1

1

1

1

1

1

1

1

1

1

1

1

1

1

1

1

1

1

1

1

1

1

1

1

1

1

1

1

1

1

1

1

1

1

1

1

1

1

1

1

1

1

1

1

1

1

1

1

1

1

1

1

1

1

1

1

1

1

1

1

1

1

1

1

1

1

1

1

1

1

1

1

1

1

1

1

1

1

1

1

1

1

1

1

1

1

1

1

1

1

1

1

1

1

1

1

1

1

1

1

1

1

1

1

1

1

1

1

1

1

1

1

1

1

1

1

1

1

1

1

1

1

1

1

1

1

1

1

1

1

1

1

1

1

1

1

1

1

1

1

1

1

1

1

1

1

1

1

1

1

1

1

1

1

1

1

1

1

1

1

1

1

1

1

1

1

1

1

1

1

1

1

1

1

1

1

1

1

1

1

1

1

1

1

1

1

1

1

1

1

1

1

1

1

1

1

1

1

1

1

1

1

1

1

1

1

1

1

1

1

1

1

1

1

1

1

1

1

1

1

1

1

1

1

1

1

1

1

1

1

1

1

1

1

1

1

1

1

1

1

1

1

1

1

1

1

1

1

1

1

1

1

1

1

1

1

1

1

1

1

1

1

1

1

1

1

1

1

1

1

1

1

1

1

1

1

1

1

1

1

1

1

1

1

1

1

1

1

1

1

1

1

1

1

1

1

1

1

1

1

1

1

1

1

1

1

1

1

1

1

1

1

1

1

1

1

1

1

1

1

1

1

1

1

1

1

1

1

1

1

1

1

1

1

1

1

1

1

1

1

1

1

1

1

1

1

1

1

1

1

1

1

1

1

1

1

1

1

1

1

1

1

1

1

1

1

1

1

1

1

1

1

1

1

1

1

1

1

1

1

1

1

1

1

1

1

1

1

1

1

1

1

1

1

1

1

1

1

1

1

1

1

1

1

1

1

1

1

1

1

1

1

1

1

1

1

1

1

1

1

1

1

1

1

1

1

1

1

1

1

1

1

1

1

1

1

1

1

1

1

1

1

1

1

1

1

1

1

1

1

1

1

1

1

1

1

1

1

1

1

1

1

1

1

1

1

1

1

1

1

1

1

1

1

1

1

1

1

1

1

1

1

1

1

1

1

1

1

1

1

1

1

1

1

1

1

1

1

1

1

1

1

1

1

1

1

1

1

1

1

1

1

1

1

1

1

1

1

1

1

1

1

1

1

1

1

1

1

1

1

1

1

1

1

1

1

1

1

1

1

1

1

1

1

1

1

1

1

1

1

1

1

1

1

1

1

1

1

1

1

1

1

1

1

1

1

1

1

1

1

1

1

1

1

1

1

1

1

1

1

1

1

1

1

1

1

1

1

1

1

1

1

1

1

1

1

1

1

1

1

1

1

1

1

1

1

1

1

1

1

1

1

1

1

1

1

1

1

1

1

1

1

1

1

1

1

1

1

1

1

1

1

1

1

1

1

1

1

1

1

1

1

1

1

1

1

1

1

1

1

1

1

1

1

1

1

1

1

1

1

1

1

1

1

1

1

1

1

1

1

1

1

1

1

1

1

1

1

1

1

1

1

1

1

1

1

1

1

1

1

1

1

1

1

1

1

1

1

1

1

1

1

1

1

1

1

1

1

1

1

1

1

1

1

1

1

1

1

1

1

1

1

1

1

1

1

1

1

1

1

1

1

1

1

1

1

1

1

1

1

1

1

1

1

1

1

1

1

1

1

1

1

1

1

1

1

1

1

1

1

1

1

1

1

1

1

1

1

1

1

1

1

1

1

1

1

1

1

1

1

1

1

1

1

1

1

1

1

1

1

1

1

1

1

1

1

1

1

1

1

1

1

1

1

1

1

1

1

1

1

1

1

1

1

1

1

1

1

1

1

1

1

1

1

1

1

1

1

1

1

1

1

1

1

1

1

1

1

1

1

1

1

1

1

1

1

1

1

1

1

1

1

1

1

1

1

1

1

1

1

1

1

1

1

1

1

1

1

1

1

1

1

1

1

1

1

1

1

1

1

1

1

1

1

1

1

1

1

1

1

1

1

1

1

1

1

1

1

1

1

1

1

1

1

1

1

1

1

1

1

1

1

1

1

1

1

1

1

1

1

1

1

1

1

1

1

1

1

1

1

1

1

1

1

1

1

1

1

1

1

1

1

1

1

1

1

1

1

1

1

1

1

1

1

1

1

1

1

1

1

1

1

1

1

1

1

1

1

1

1

1

1

1

1

1

1

1

1

1

1

1

1

1

1

1

1

1

1

1

1

1

1

1

1

1

1

1

1

1

1

1

1

1

1

1

1

1

1

1

1

1

1

1

1

1

1

1

1

1

1

1

1

1

1

1

1

1

1

1

1

1

1

1

1

1

1

1

1

1

1

1

1

1

1

1

1

1

1

1

1

1

1

1

1

1

1

1

1

1

1

1

1

1

1

1

1

1

1

1

1

1

1

1

1

1

1

1

1

1

1

1

1

1

1

1

1

1

1

1

1

1

1

1

1

1

1

1

1

1

1

1

1

1

1

1

1

1

1

1

1

1

1

1

1

1

1

1

1

1

1

1

1

1

1

1

1

1

1

1

1

1

1

1

1

1

1

1

1

1

1

1

1

1

1

1

1

1

5

1

1

1

1

1

1

1

1

1

1

1

1

1

1

1

1

1

1

1

1

1

1

1

1

1

1

1

1

1

373

373

1

1

1

1

1

1

1

1

1

1

1

1

1

1

1

1

1

1

1

1

1

1

1

1

1

1

1

1

1

1

1

1

1

1

1

1

1

1

1

1

1

1

1

1

1

1

1

1

1

1

1

1

1

1

1

1

1

1

1

1

1

1

1

1

1

1

1

1

1

1

1

1

1

1

1

1

1

1

1

1

1

1

1

1

1

1

1

1

1

1

1

1

1

1

1

1

1

1

1

1

1

1

1

1

1

1

1

1

1

1

1

1

1

1

1

1

1

1

1

1

1

1

1

1

1

1

1

1

1

1

1

1

1

1

1

1

1

1

1

1

1

1

1

1

1

1

1

1

1

1

1

1

1

1

1

1

1

1

1

1

1

1

1

1

1

1

1

1

1

1

1

1

1

1

1

1

1

1

1

1

1

1

1

1

1

1

1

1

1

1

1

1

1

1

1

1

1

1

1

1

1

1

1

1

1

1

1

1

1

1

1

1

1

1

1

1

1

1

1

1

1

1

1

1

1

1

1

1

1

1

1

1

1

1

1

1

1

1

1

1

1

1

1

1

1

1

1

1

1

1

1

1

1

1

1

1

1

1

1

1

1

1

1

1

1

1

1

1

1

1

1

1

1

1

1

1

1

1

1

1

1

1

1

1

1

1

1

1

1

1

1

1

1

1

1

1

1

1

1

1

1

1

1

1

1

1

1

1

1

1

1

1

1

1

1

1

1

1

1

1

1

1

1

1

1

1

1

1

1

1

1

1

1

1

1

1

1

1

1

1

1

1

1

1

1

1

1

1

1

1

1

1

1

1

1

1

1

1

1

1

1

1

1

1

1

1

1

1

1

1

1

1

1

1

1

1

1

1

1

1

1

1

1

3

3

1

1

1

1

1

1

1

1

1

1

1

1

1

1

1

1

1

1

1

1

1

1

1

1

1

1

1

1

1

1

1

1

1

1

1

1

1

1

1

1

1

1

1

1

1

1

1

1

1

1

1

1

1

1

1

1

1

1

1

1

1

604

1

1

1

252

252

1

1

1

1

1

1

1

1

1

1

1

1

1

1

1

1

1

1

1

1

1

1

1

1

1

1

1

1

1

1

1

1

1

1

1

1

1

1

1

1

1

1

1

1

1

1

1

1

1

1

1

1

1

1

1

1

1

1

1

1

1

1

1

1

1

1

1

1

1

1

1

1

1

1

1

1

1

1

1

1

1

1

1

1

1

1

1

1

1

1

1

1

1

1

1

1

1

1

1

1

1

1

1

1

1

1

1

1

1

1

1

1

1

1

1

1

1

1

1

1

1

1

1

1

1

1

1

1

1

1

1

1

1

1

1

1

1

1

1

1

1

1

1

1

1

1

1

1

1

1

1

1

1

1

1

1

1

1

1

1

1

1

1

1

1

1

1

1

1

1

1

1

1

1

1

1

1

1

1

1

1

1

1

1

1

1

1

1

1

1

1

1

1

1

1

1

1

1

1

1

1

1

1

1

1

1

1

1

1

1

1

1

1

1

1

1

1

1

1

1

1

1

1

1

1

1

1

1

1

1

1

1

1

1

1

1

1

1

1

1

1

1

1

1

1

1

1

1

1

1

1

1

1

1

1

1

1

1

1

1

1

1

1

1

1

1

1

5

1

1

4

1

1

1

1

1

1

1

1

1

1

1

1

1

1

1

1

1

1

1

1

1

1

1

44

44

1

1

1

1

1

1

1

1

1

1

1

1

1

1

1

1

1

1

1

1

1

1

1

1

1

1

1

1

1

1

1

1

1

1

1

1

1

1

1

1

1

1

1

1

20

3

1

1

1

2

1

1

3

1

1

1

2

1

1

10

1

1

1

1

1

1

1

1

1

1

1

191

1

1

1

1

1

1

1

75

1

1

1

1

1

1

1

1

1

1

1

1

1

1

1

1

1

1

1

1

1

1

1

1

1

1

1

1

1

1

1

1

1

1

1

1

1

1

1

1

1

1

1

1

1

1

1

1

1

1

1

1

1

1

1

1

1

1

1

1

1

1

1

1

1

1

1

1

1

1

1

1

1

1

1

106

1

1

1

1

1

1

1

1

1

1

1

1

1

1

1

1

1

1

1

1

1

1

1

1

1

1

1

1

1

1

1

1

1

1

1

1

1

1

1

1

1

1

1

1

1

1

1

1

1

1

1

1

1

1

1

1

1

1

1

1

1

1

1

1

1

1

1

1

1

1

1

1

1

1

1

1

1

1

1

1

1

1

1

1

1

1

1

1

1

1

1

1

1

1

1

1

1

1

1

1

1

1

1

1

1

1

3

1

1

1

1

1

1

23

1

1

1

3

1

1

1

1

1

1

1

12

1

1

1

1

1

1

1

1

1

1

1

1

1

1

1

1

1

28

28

1

1

1

1

1

1

1

1

1

1

1

1

1

1

1

1

1

1

1

1

1

1

1

1

1

1

1

1

1

2

2

1

1

1

1

1

1

1

1

1

1

1

1

1

1

1

1

1

1

1

1

1

1

1

1

1

1

1

1

1

1

1

1

1

1

1

1

1

1

1

2

2

2

1

1

1

3

3

3

3

1

1

1

1

1

1

1

1

1

1

1

1

1

11

11

11

11

1

1

1

1

1

1

1

1

1

1

1

1

1

1

1

1

1

1

1

1

1

1

5

2

2

2

1

1

2

2

2

1

1

1

1

1

1

1

1

1

1

1

1

1

1

1

1

1

5

5

3

3

1

1

1

2

2

1

1

1

1

1

1

21

21

21

15

1

1

1

1

1

1

1

1

1

1

1

1

1

1

1

1

1

1

1

1

1

3

1

1

1

1

1

1

1

1

1

1

1

1

1

1

1

131

69

4

3

1

1

1

1

1

59

59

1

1

1

1

1

1

1

1

1

1

1

1

1

1

1

1

1

1

1

1

1

1

1

1

1

1

1

1

1

1

1

1

1

1

1

1

1

1

1

1

1

1

1

1

1

1

1

1

1

1

1

1

1

1

1

1

1

1

1

6

6

1

1

1

1

1

1

62

1

1

1

61

61

1

1

1

1

1

1

1

1

1

1

1

1

1

1

1

1

1

1

1

1

1

1

1

1

1

1

1

1

1

1

1

1

1

1

1

1

1

1

1

1

1

1

1

1

1

1

1

1

1

1

1

1

1

1

1

1

1

1

1

1

1

162

162

162

162

1

1

1

1

1

1

1

1

1

1

1

1

1

1

1

1

1

1

1

1

1

1

1

1

1

1

1

1

1

1

1

1

1

1

1

1

1

1

1

1

1

1

1

1

1

1

1

1

1

1

1

1

1

1

1

1

1

1

1

1

1

1

1

1

1

1

1

1

1

1

1

1

1

1

1

1

1

1

1

1

1

1

1

1

1

1

1

1

1

1

1

1

1

1

1

1

1

1

1

1

1

1

1

1

1

1

1

1

1

1

1

1

1

1

1

1

1

1

1

1

1

1

1

1

1

1

1

1

1

1

1

1

1

1

1

1

1

1

1

1

1

1

1

1

1

1

1

1

1

1

1

1

1

1

1

1

1

1

1

1

1

1

1

1

1

1

1

1

1

1

1

1

1

1

1

1

1

1

1

1

1

1

1

1

1

1

1

1

1

1

1

1

1

1

1

1

1

1

1

1

1

1

1

1

1

1

1

1

1

1

1

1

1

1

1

1

1

1

1

1

1

1

1

1

1

1

1

1

1

1

1

1

1

1

1

1

1

1

1

1

1

1

1

1

1

1

1

1

1

1

1

1

1

1

1

1

1

1

1

1

1

1

1

1

27

20

20

1

1

1

1

17

1

1

1

1

1

1

1

1

1

1

1

1

1

1

1

1

1

7

3

3

1

1

1

3

3

1

1

1

1

1

1

3

1

1

1

1

2

2

2

1

1

1

1

1

1

1

1

1

1

1

149

3

2

1

1

1

1

1

1

1

7

3

1

1

2

1

1

4

4

1

1

1

1

41

11

11

1

1

1

1

1

1

1

1

1

1

1

3

3

1

1

1

6

1

1

1

1

4

1

1

1

1

11

2

1

1

8

1

1

1

1

1

1

1

1

1

1

3

3

1

1

1

6

1

1

3

1

1

1

2

1

1

1

1

1

97

1

96

1

1

1

52

1

1

1

1

1

1

1

1

1

1

1

1

1

1

1

1

1

1

1

1

1

1

1

1

1

1

1

1

1

1

1

1

1

1

1

1

1

1

1

1

1

1

1

1

1

1

1

1

1

1

1

1

2

1

1

1

23

1

1

1

1

1

1

1

1

1

1

1

1

1

1

1

1

1

1

1

1

1

1

1

7

1

1

1

1

1

1

1

6

1

1

1

1

1

1

1

1

1

1

1

1

368

1

184

3

3

1

1

1

9

1

1

1

1

4

1

1

1

1

3

1

1

1

9

7

1

1

1

1

1

1

1

1

1

1

1

1

1

1

1

1

4

2

1

1

2

1

1

1

1

1

3

3

1

1

1

11

11

1

1

1

1

1

1

1

1

1

1

1

1

1

1

1

1

1

1

5

5

1

1

1

1

1

1

1

1

1

1

1

1

1

1

1

1

63

1

1

1

1

4

1

1

1

1

1

1

4

1

1

1

1

2

1

1

1

1

1

1

1

1

1

1

1

4

1

1

1

1

1

1

38

1

1

1

1

1

1

1

1

1

1

1

1

1

1

1

1

1

1

1

1

1

1

1

1

1

1

1

1

1

1

1

1

1

1

1

1

1

1

1

6

6

1

1

1

1

1

1

1

1

1

2

2

1

1

14

3

1

1

1

11

1

1

1

1

1

1

1

1

1

1

1

1

1

1

3

3

1

1

1

6

1

3

1

1

1

1

1

1

1

2

2

1

1

1

1

1

1

6

6

1

1

1

1

1

1

1

7

1

1

1

1

2

1

1

3

1

1

1

1

1

1

1

5

5

1

1

1

1

1

1

1

2

2

1

1

1

1

1

1

1

1

179

1

1

1

1

1

1

1

1

1

1

1

28

1

1

26

1

1

1

1

1

1

1

1

1

1

1

1

1

1

1

1

1

1

1

1

1

1

1

1

1

1

1

1

1

6

4

1

1

1

1

2

1

1

1

1

2

2

1

1

1

1

1

13

13

1

1

1

1

1

1

1

1

1

1

1

1

1

2

2

1

1

1

1

1

65

51

1

1

1

1

1

1

1

1

1

1

1

1

1

1

1

1

1

1

1

1

1

1

1

1

1

1

1

1

1

1

1

1

1

1

1

1

1

1

1

1

1

1

1

1

1

1

1

1

1

1

1

1

1

1

4

1

1

1

1

1

1

4

1

1

1

1

1

1

1

1

1

1

1

1

1

1

1

1

1

1

1

1

3

1

1

1

1

1

1

1

17

1

1

5

1

1

1

1

1

5

1

1

1

1

1

1

1

1

1

2

1

1

2

1

1

1

1

1

1

4

4

1

1

1

1

1

1

1

1

1

1

1

1

2

1

1

1

1

1

1

1

1

1

1

1

1

1

1

1

1

1

1

1

1

13

9

1

1

1

3

3

1

1

1

4

3

1

1

1

1

1

1

1

1

2

1

1

1

1

1

1

1

1

1

1

1

1
